# Identification Of Endothelial Cell Immune-related Gene Signature for Lung Adenocarcinoma by Integrated Analysis of Single-cell and Bulk RNA Sequencing Data

**DOI:** 10.7150/jca.94501

**Published:** 2024-05-20

**Authors:** Zhuozheng Hu, Jiajun Wu, Weijun Zhou, Kang Wang, Wenxiong Zhang

**Affiliations:** 1 Department of Thoracic Surgery, The Second Affiliated Hospital, Jiangxi Medical College, Nanchang University, Nanchang, 330006, China.; 2 Department of Traditional Chinese Medicine, The Second Affiliated Hospital, Jiangxi Medical College, Nanchang University, Nanchang, 330006, China.

**Keywords:** Endothelial cell, Immune-related genes, Lung adenocarcinoma, Prognosis Signature, Single-Cell, Bulk RNA-Sequencing

## Abstract

**Background:** The role of endothelial cells in tumor progression is considerable, yet the effect of endothelial cell immune-related genes (EIRGs) is still unclear. This research aimed to scrutinize the prognostic value of EIRGs in lung adenocarcinoma (LUAD) and provide further insights into the abovementioned uncertainties.

**Methods:** After single-cell RNA sequencing (scRNA-seq) samples were obtained from the Gene Expression Omnibus (GEO) database, they were integrated with bulk RNA sequencing data from The Cancer Genome Atlas (TCGA). Prognostic markers were determined and a prognostic model was developed. From this model, a nomogram was constructed. We analyzed the biological mechanism of the EIRGs in LUAD, including functional enrichment, tumor mutational burden (TMB), tumor microenvironment (TME) analyses and drug sensitivity. We validated the signature by validating the external cohort GSE31210 and RT-qPCR.

**Results:** After analyzing the model constructed from eight EIRGs, we observed that high-risk group (HG) LUAD patients (a risk score exceeding 4.65) exhibited unfavorable outcomes according to Kaplan‒Meier survival curves. This outcome was confirmed by GSE31210. The nomogram based on the model demonstrated significant predictive value. HG was influenced primarily by steroid hormone biosynthesis and ECM receptor interactions. The TMB in HGs was greater than that in the LG. Analysis of drug sensitivity revealed the direction for individualized treatment for both risk cohorts. Variations in the expression of EIRGs have been confirmed via RT-qPCR in several LUAD cell lines.

**Conclusions:** The prognostic model and nomogram above are valuable for determining the survival rate and treatment options for LUAD patients.

## Introduction

Lung cancer ranks among the most common malignant tumors worldwide and is characterized by a high occurrence and fatality rate. LUAD is one of the major pathological types of lung cancer 1, 2. Accurate prognostic assessment of patients with LUAD can strongly influence the treatment of LUAD. Currently, the widely adopted tumor node metastasis (TNM) classification categorizes patients into stages I through IV and uses this system to estimate patient prognosis 3. However, this conventional classification method fails to accurately predict the prognosis of some LUAD patients. Biomarkers have recently been utilized to construct models that forecast patient prognosis. Hence, there is an immediate need to explore dependable biomarkers to create innovative signatures for classifying the risk of LUAD patients, ultimately resulting in a more precise evaluation of patient prognosis.

The TME relies on microvascular endothelial cells to regulate immune surveillance and provide cancer cells with nutrients and oxygen. Nevertheless, as a result of the disarray of tumor microvascular tissue, the blood vessels in developing tumors become hypoperfusion and excessively permeable 4. Consequently, cancer cells are unable to adequately gain oxygen and generate oxygen, and the immune response in the TME becomes insufficient 5. This effect is undoubtedly beneficial for ensuring the resistance of tumor cells to treatment. As a result, the prognosis of patients may be linked to EIRGs. With the emergence of scRNA-seq technology, the analysis of various cell types at the individual cell level has become feasible. For example, a prognostic model based on 4 hub genes of endothelial cells was created to predict the prognosis of patients with glioblastoma multiforme (GBM) 6. The survival rate of LUAD patients can be forecasted by confirming the model built using 5 T-cell marker genes 7. The above models are better than the traditional staging system for predicting LUAD patients. However, a predictive model for LUAD based on EIRGs is still lacking.

Through the analysis of the integration of scRNA-seq and bulk RNA sequencing data, a prognostic model and nomogram were constructed. In addition, we performed enrichment analysis and TME analysis to explore the underlying molecular mechanism involved.

## Materials and methods

### Data sources and access

For our study, the scRNA-seq data of three LUAD samples (GSE117570) were acquired. The bulk sequencing, mutation, and characteristic data of 522 LUAD patients were obtained from the TCGA database (normalized to FPKM), samples with any unknown characteristic data were excluded. In addition, we downloaded the GSE31210 dataset, and the model accuracy was verified using the GSE31210 dataset as an independent external cohort. Immune-related genes were derived from the Innatedb website (https://www.innatedb.ca/).

### Processing and analysis of the source data

The 'Seurat' package in R software was utilized to convert the scRNA-seq data into seurat objects 8. To ensure quality control, the following parameters were set: cell counts were less than 3, cells were less than 50 genes mapped, and cells were considered to have mitochondrial genes comprising more than 5%. The "NormalizeData" function was then applied to standardize the data after quality control. A total of 1,500 genes that had a substantial coefficient of variation were subsequently selected. Using the "ScaleData" function, 20 principal components were extracted through principal component analysis (PCA) after preprocessing these genes. Finally, the P values for each of these primary components were ascertained through the use of the "JackStraw" function. We classified the unknown cell subgroup according to the functions "FindNeighbors" and "FindClusters" (resolution=0.5). Subsequently, t-distributed stochastic neighbor embedding (tSNE) was applied to visualize and unsupervised clustering the above outcome 9. The "FindAllMarkers" package was used to detect differentially expressed genes (DEGs) in every cluster, for which logFC=1 (differential multiples) and the expression ratio of the least differential genes =0.25; these particular genes are referred to as DEGs. Cell types were annotated using the "SingleR" package 10.

### Screening of EIRGs

The DEGs of endothelial cells were filtered out from all DEGs using R software. After that, we used the "VennDiagram" function to obtain the EIRGs of the endothelial cell DEGs and immune-related genes for subsequent analysis.

### Functional enrichment analysis and protein‒protein interaction (PPI) analysis

We utilized the "clusterProfiler" tool for analyzing both Gene Ontology (GO)^11^ and Kyoto Encyclopedia of Genes and Genomes (KEGG)^12^ data to investigate key enrichment pathways and biological processes of the EIRGs. A PPI map of the constructed EIRGs was constructed via the Search Tool for the Retrieval of Interacting Genes/Proteins (STRING) website (https://cn.string-db.org/).

### Construction of the prognostic model

Univariate Cox regression analysis was performed to obtain EIRGs with significant prognostic correlation. On the basis of the "glmnet" package in R software, least absolute shrinkage and selection operator (LASSO) regression was conducted to reduce the number of model genes 13. The risk score was calculated as follows: risk score = coefficient (Gene 1) × expression (Gene 1) + coefficient (Gene 2) × expression (Gene 2) + coefficient (Gene 3) × expression (Gene 3) +......+ coefficient (Gene n) × expression (Gene n). Based on the median risk score, all the TCGA samples were split into two groups. The "SurvivalROC" function was used to construct receiver operating characteristic (ROC) curves and area under the curve (AUC) values to evaluate the precision of the model's ability to predict LUAD prognosis in patients with 1-, 3-, and 5-year OS. Independent cohort validation of the signature was subsequently performed using the GSE31210 dataset to test the signature's predictive ability.

### Nomogram construction and clinical relevance analysis

To construct a nomogram and evaluate its accuracy, we utilized the 'RMS' package in R software, incorporating age, gender, stage, and risk 14. Additionally, calibration curves were created to measure the precision of the nomogram. In our study, we combined risk scores with sample characteristics and probed their clinical relevance. To illustrate this, we generated correlation heatmaps according to the "ComplexHeatmap" function in R software.

### Enrichment analysis

Through the functions "limma", "org.Hs.eg.db", "clusterProfiler" and "enrichplot", gene set enrichment analysis (GSEA)^15^ was performed to explore pathways and molecular mechanisms of notable enrichment.

### Tumor mutational burden

For the otherness of the genetic mutations between the two groups, we utilized the TCGA database for the analysis of somatic mutation data in tumors. The "maftools" package from R software was subsequently utilized to create two waterfall plots. The "ggpubr" package in R software was used to determine the difference in TMB between different risk groups.

### Tumor immune infiltration status analysis

Using the CIBERSORT technique, with 1,000 permutations, the immune microenvironment was studied to ascertain the composition of 22 distinct immune cells 16. The stromal, ESTIMATE, and immune scores for the various risk groups were calculated using the "ESTIMATE" package 17. The above data were also visualized in a violin plot.

### Analysis of drug sensitivity and the response to immunotherapy

In our research, we investigated the associations between various risk levels and genes associated with immune checkpoints. To predict the impact of immunotherapy in both groups, the Tumor Immune Dysfunction and Exclusion (TIDE) algorithm (http://tide.dfci.harvard.edu/) was applied 18. Using the R package "oncoPredict", we were able to predict the susceptibility of various groups to chemotherapy drugs.

### Cell culture and RT‒qPCR

We derived lung epithelial cell lines of normal origin (BEAS-2B) and LUAD cell lines (H1395 and H1975) from the Typical Culture Preservation Commission Cell Bank of the Chinese Academy of Medicine Sciences (Shanghai, China). Medium containing epithelial cell growth factor (BEGM Kit, LONZA Corporation, USA) was used to culture normal lung epithelial cells. Cancer cell lines (H1395, H1975, and A549) were cultured in RPMI 1640 medium supplemented with 10% fetal bovine serum and 1% penicillin‒streptomycin (BioLegend Kit). Total RNA was extracted from the cell lines using TRIzol reagent (Takara Bio, Inc., Otsu, Japan) according to the manufacturer's instructions. cDNA was synthesized using a reverse transcription kit from Accurate Biology (Hunan, China), and the SYBR Green premixed qPCR kit (Accurate Biology, Hunan, China) was used in a Roche LightCycler 480 II (Roche, Basel, China). Subsequently, RT‒qPCR was performed. Using the 2-ΔΔCt technique, comparative expression levels of each gene were determined. **Table S1** contains a comprehensive list of the complete primer sequences.

We used the Human Protein Atlas database (https://www.proteinatlas.org/) to compare the protein expression levels of the EIRGs between LUAD tissues and normal tissues.

### Statistical analysis

Our analysis of the data was conducted with R software version 4.2.3 and Perl language (Strawberry Perl 5.30.0.1). The Wilcoxon t test was applied for the comparison of variables across both groups, and a p value less than 0.05 was considered to indicate a significant difference.

## Results

### Identification of EIRGs

**Figure 1:**
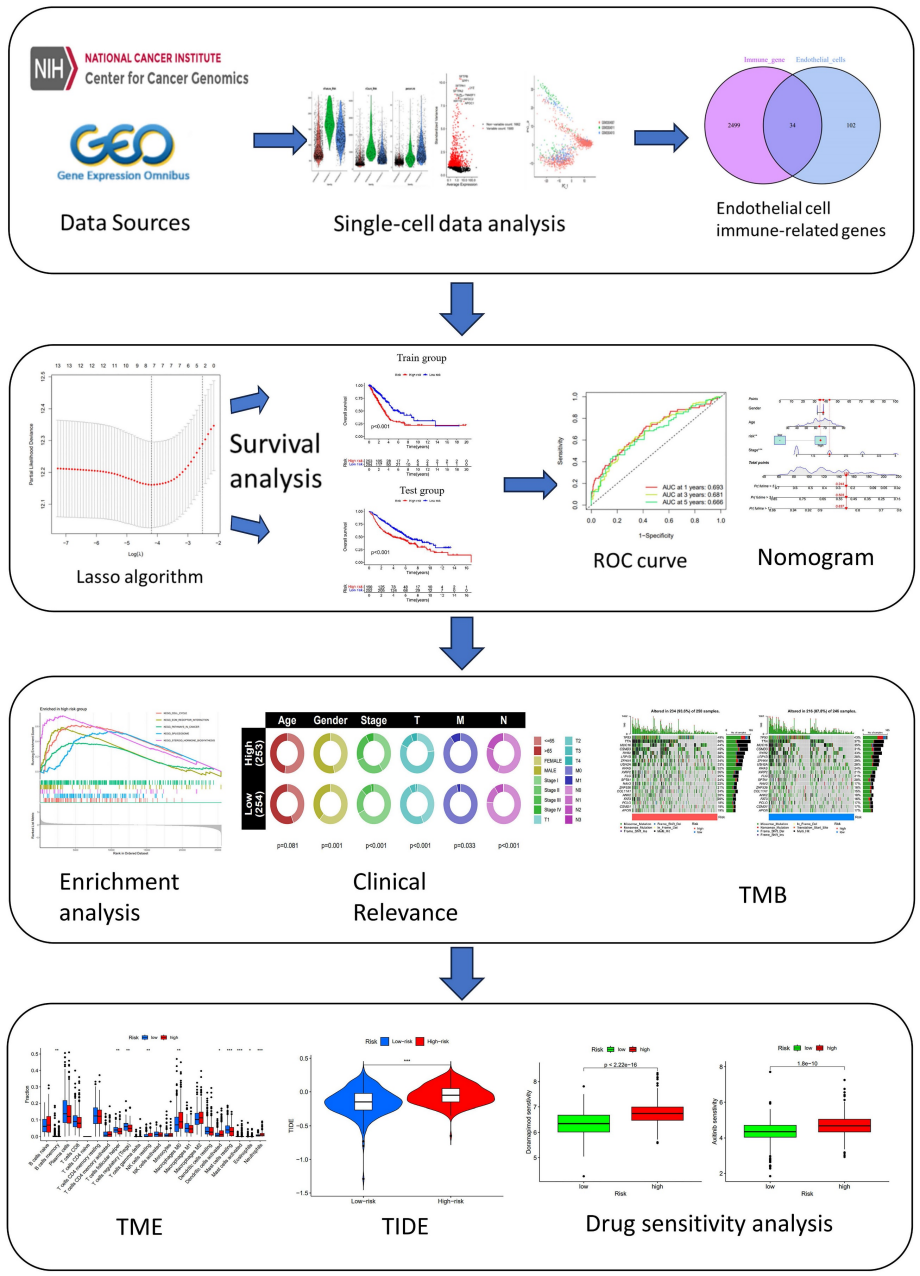
Flow Chart.

**Figure 2:**
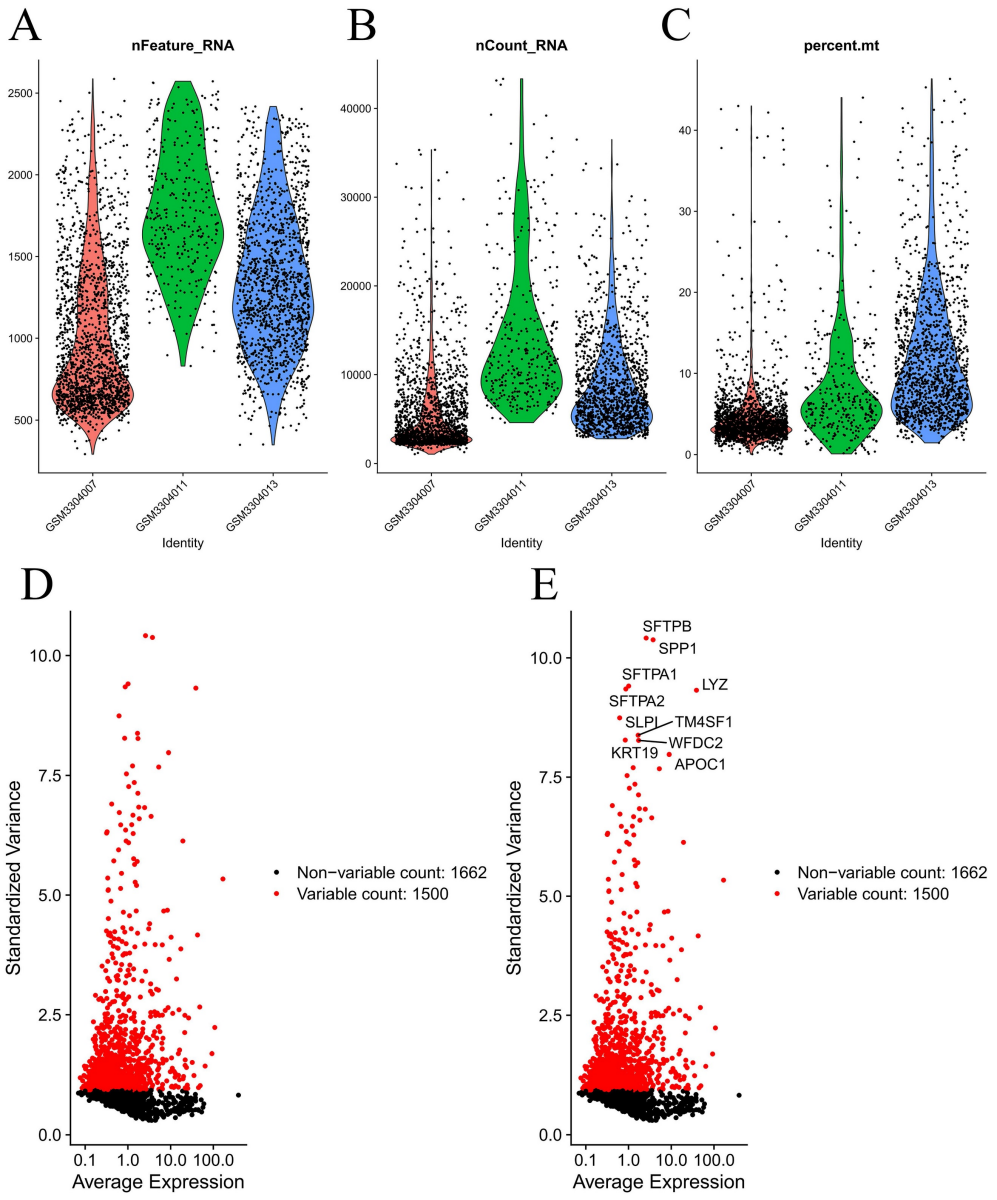
Processing of raw data. Quality control of scRNA-seq data from three LUAD samples (A-C); The variance plot showed 1662 genes in all cells, red dots represent the top 1500 highly variable genes (D-E).

**Figure 3:**
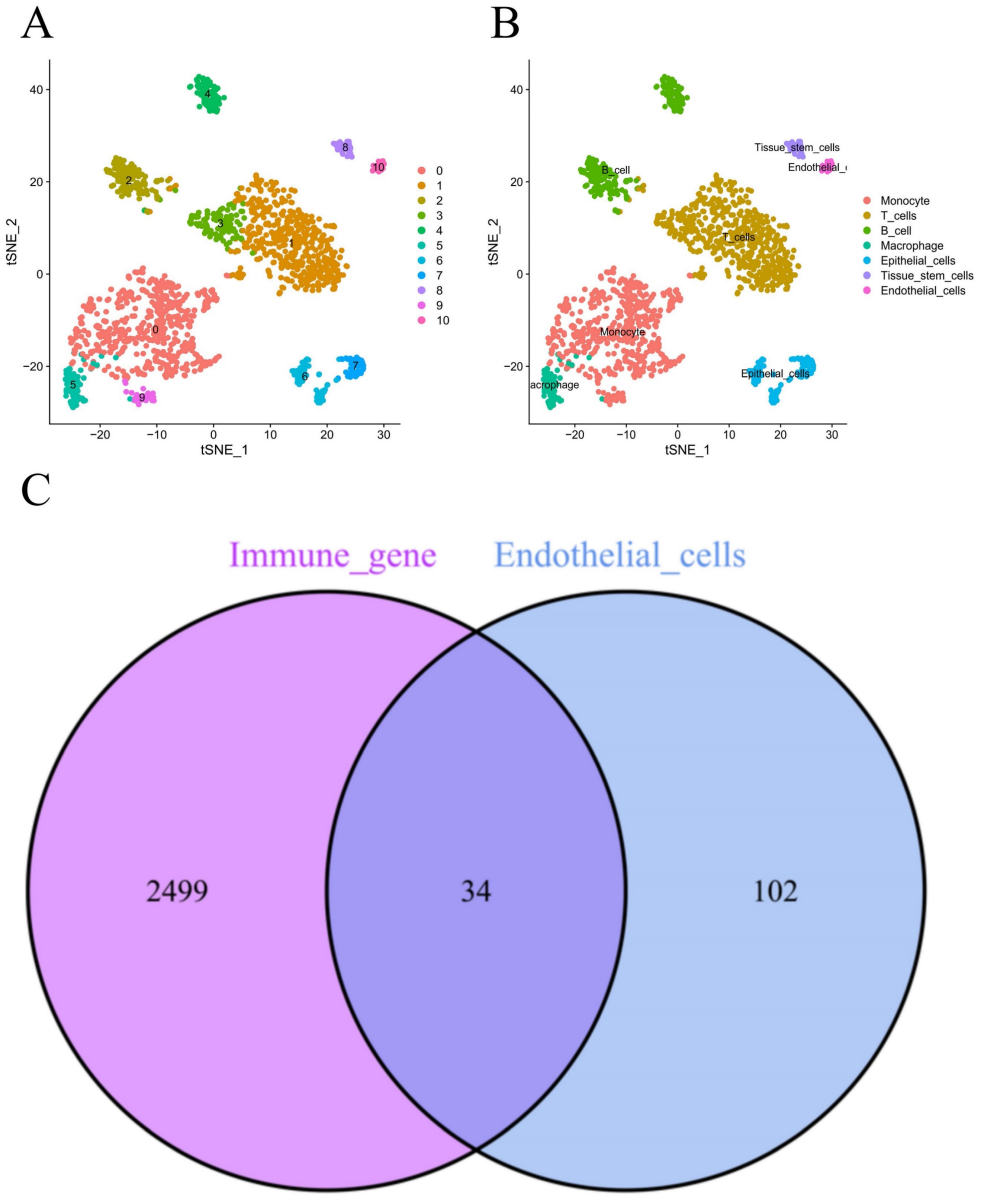
Unsupervised clustering and obtain for EIRGs. 11 clusters were visualized based on the t-SNE algorithm (A); Cell subpopulations identified (B); Acquisition of endothelial cell immune-related genes (C).

**Figure 1** demonstrates the process of this research. We obtained scRNA-seq data from three LUAD samples within the GSE117570 dataset in this research. For quality control and filtering, the gene count, sequencing depth, and proportion of mitochondria in the three samples were evaluated, and we subsequently acquired 1,695 cells **(Figure 2A-C)**. After standardizing the data, a total of 1500 genes with significant variation were chosen **(Figure 2D-E)**, and 20 principal components (P<0.05) were selected for analysis following dimensionality reduction via principal component analysis (PCA) **(Figure S1A)**. The cells were clustered and visualized using the tSNE algorithm, and the cell subpopulations were annotated using the "singleR" function. The heatmap shows the expression levels of marker genes in each cluster **(Figure S1B)**. In our investigation, we discovered that there were 11 clusters and 7 different cell types present in the cells **(Figure 3A)**. Notably, Cluster 10 represented the endothelial cell subpopulation, as depicted in** Figure 3B**. Next, we identified DEGs in endothelial cells **(Table S2)**. After obtaining the DEGs of endothelial cells, the genes were intersected with the immune-related genes, and 34 EIRGs were obtained **(Figure 3C)**.

### Functional enrichment analysis and protein‒protein interaction (PPI) analysis

GO analysis results **(Figure S2A-C)** indicated that biological process (BP) was enriched in mainly viral process and cytokine-mediated processes. Among the cellular component (CC) terms, the pathways were enriched in signaling pathways and cell-substrate junctions. The main enrichment pathway of molecular functions (MF) was cytokine binding. The KEGG analysis results **(Figure S3A-B)** revealed Kaposi sarcoma-associated herpesvirus infection, the TNF signaling pathway, human immunodeficiency virus 1 infection and human T-cell leukemia virus 1 infection. According to the GO analysis results, the mechanism of action mainly involves the conduction and reception of cytokines. The results of the KEGG analysis were mainly related to the destruction of the immune system. We performed a protein‒protein interaction analysis based on the EIRGs, minimum required interaction score>0.4 and hidden disconnected nodes in the network **(Figure S4A)**. We found that PTPRC had the highest number of junction nodes and was the most likely core gene of the network **(Figure S4B)**.

### Building and validating the prognostic model

**Figure 4:**
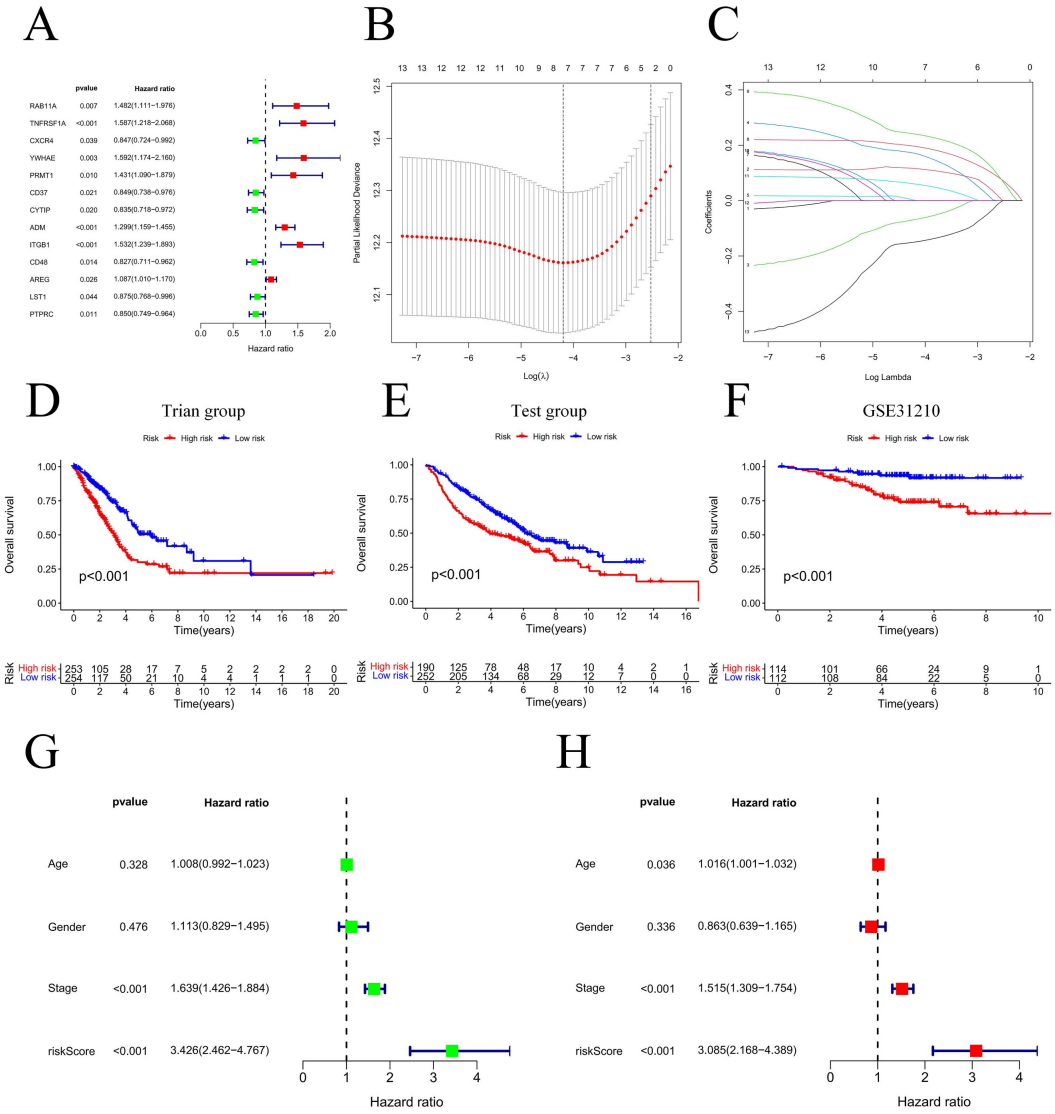
Model construction and prognosis analysis. Forest plot of multivariate Cox regression result (A); LASSO regression analysis (B, C); Compare the survival of the high-risk group and low-risk group (D, E); Compare the survival of the high-risk group and low-risk group on the GSE31210 (F); Univariate and multivariate Cox regression (G, H). * P<0.05.

**Figure 5:**
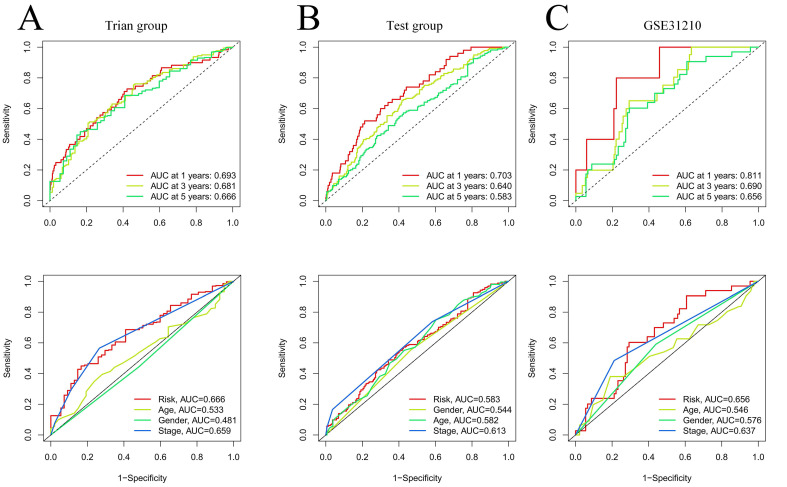
Validation of model effects. The AUC at 1-, 3-, 5-years and different clinical information of prognostic models in the train cohort (A); The AUC at 1-, 3-, 5-years and different clinical information of prognostic models in the test cohort (B); The AUC at 1-, 3-, 5-years and different clinical information of prognostic models in the GSE31210 (C).

Our team applied univariate Cox regression analysis based on 34 intersecting genes and identified 13 biomarkers that could be used to construct the model **(Figure 4A) (Table S3)**. Then, according to the R software package "glmnet", LASSO regression was used to further reduce the biomarker count, as depicted in **Figure 4B-C**. Through this process, eight EIRGs were identified, namely, TNFRSF1A, CXCR4, YWHAE, PRMT1, ADM, ITGB1, AREG, and PTPRC. The risk score formula of the model, represented by their coefficients, can be expressed as follows: risk score = (0.116 × TNFRSF1A expression) + (-0.068 × CXCR4 expression) + (0.170 × YWHAE expression) + (0.001 × PRMT1 expression) + (0.196 × ADM expression) + (0.240 × ITGB1 expression) + (0.060 × AREG expression) + (-0.149 × PTPRC expression) **(Figure S5A-H) (Table S4)**. For in-depth analysis, we utilized the TCGA cohort as the train cohort, whereas the GEO cohort served as the test cohort **(Table 1)**. When patients were classified into the HG or LG according to the median risk score, the latter cohort had a more extended OS than the former **(Figure 4D-E) (Table 2)**. Analysis of the external independent cohort GSE31210 yielded comparable outcomes** (Figure 4F)**. Our study findings were based on univariate and multivariate Cox regression analyses of age, gender, stage, and risk score in TCGA LUAD patients; the results are shown in **Figure 4G-H** and **Table S5**. Stage and risk score were found to be separate outcome factors. The area under the curve (AUC) from **Figure 5A** indicates that the train cohort had survival rates of 0.693, 0.681, and 0.666 at 1, 3, and 5 years, respectively. Conversely, the AUCs for 1, 3, and 5 years were 0.703, 0.640, and 0.583, respectively, as shown in **Figure 5B**. In addition, we employed the GSE31210 cohort as an independent group to verify the precision of the prognostic signature, with 0.811, 0.690, and 0.656 at 1, 3, and 5 years, respectively **(Figure 5C)**. To validate the model's predictive value and obtain a more intuitive graphical representation, we generated heatmaps illustrating the expression of EIRGs in various risk groups **(Figure S6A-B)**. The expression of TNFRSF1A, CXCR4, YWHAE, PRMT1 and ITGB1 was upregulated in the HG, and the test cohort yielded results consistent with those of the train cohort. The risk distribution plot **(Figure S6C-D)** and risk curves were also consistent with our predicted results **(Figure S6E-F)**.

### Nomogram construction and clinical relevance analysis

**Figure 6:**
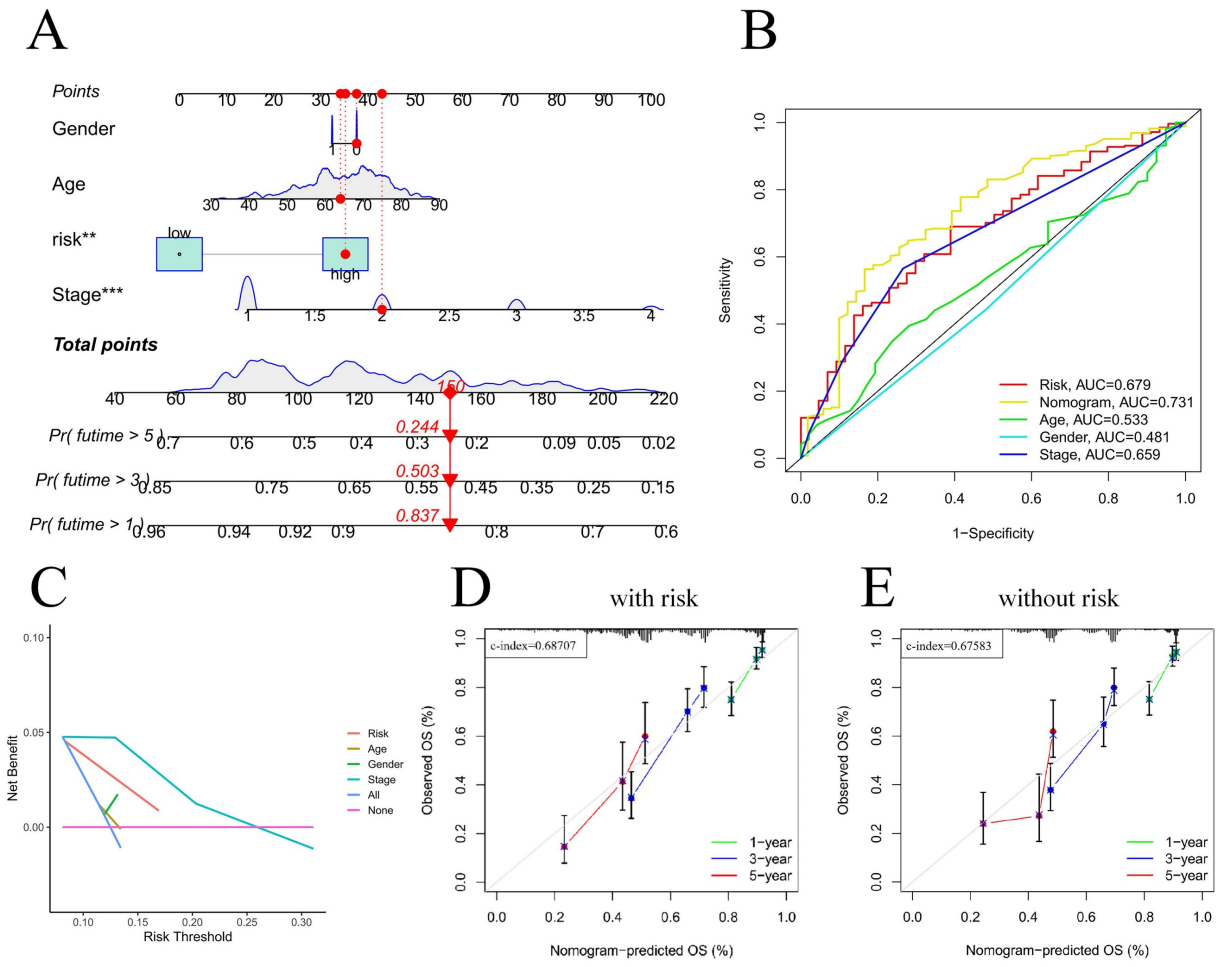
Nomogram predicts patient prognosis. Nomogram predicts patient prognosis (A); ROC curves containing different clinical information and nomogram (B); Decision curve to test for forecast value (C); Calibration curve with risk (D); Calibration curve without risk (E).

**Figure 7:**
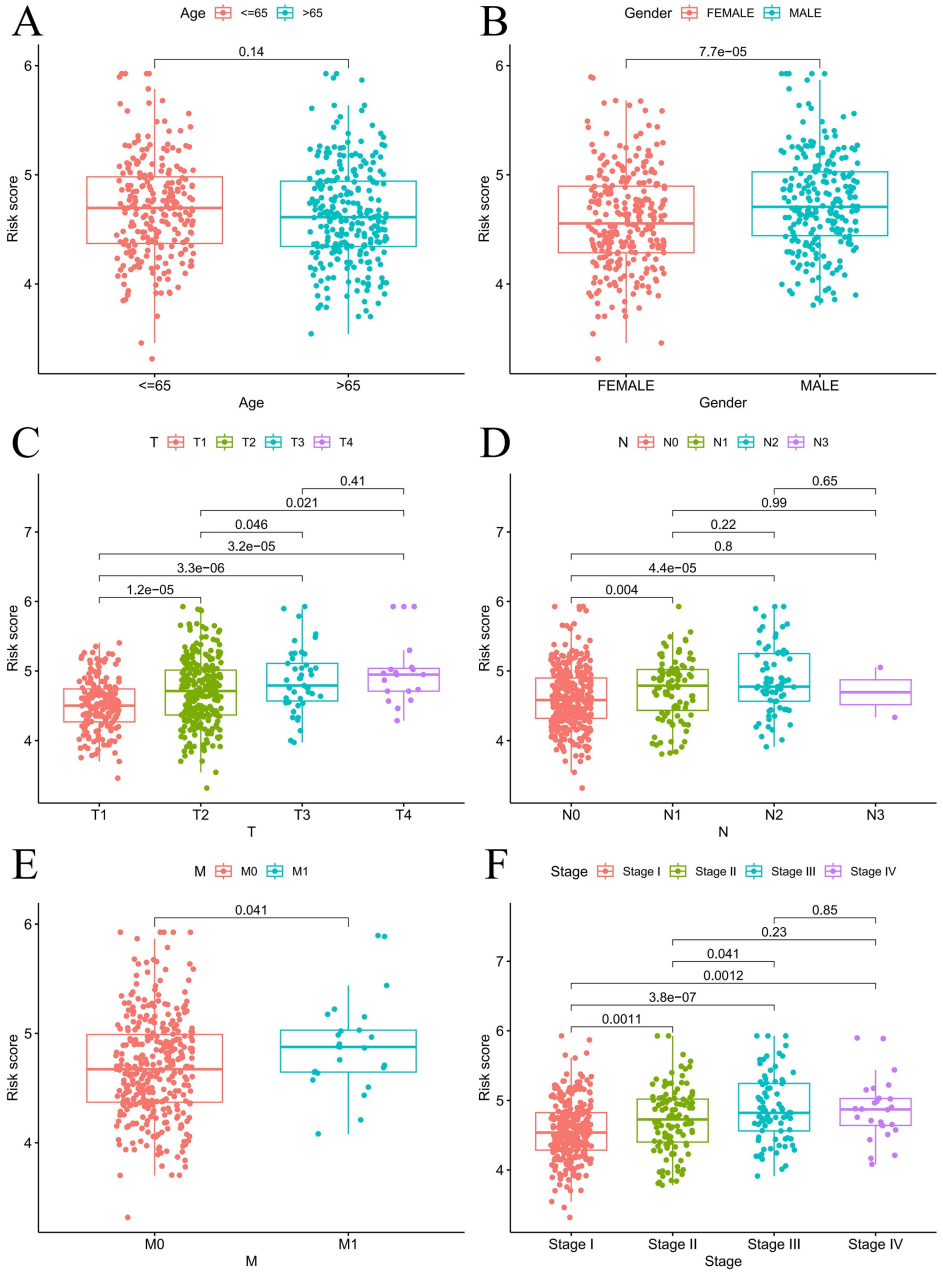
Prognosis analysis under different clinical characteristics. Boxplot of risk scores based on EIRGs signature with different clinical information (A-F).

For the prognostic evaluation of TCGA patients **(Figure 6A)**, we developed a nomogram utilizing the TCGA dataset and considering variables such as age, gender, stage, and risk score. ROC curve and decision curve analyses demonstrated that risk had superior predictive efficacy compared to alternative clinical characteristics **(Figure 6B-C)**. The calibration curves in **Figure 6D-E** indicate that the risk model (C-index=0.68707) exhibited enhanced predictive ability in contrast to the nomogram without risk (C-index=0.67583). We also conducted a clinical relevance analysis for both groups. Notably, there were associations between the risk score and various clinical features, including stage, T stage, and N stage **(Figure S7A-B)**. Afterward, we individually assessed the association between each of these features and the risk score. The risk scores of males were notably greater than those of females **(Figure 7B)**. The risk score increased significantly with increasing T stage **(Figure 7C)**, and the correlation between N stage and risk score was also consistent with the above findings **(Figure 7D)**. The risk score of M1 was greater than that of M0** (Figure 7E)**. The correlation between the progression stage and the increase in the risk score was also substantial, as illustrated in **Figure 7F**.

### Enrichment analysis between the high- and low-risk groups

**Figure 8:**
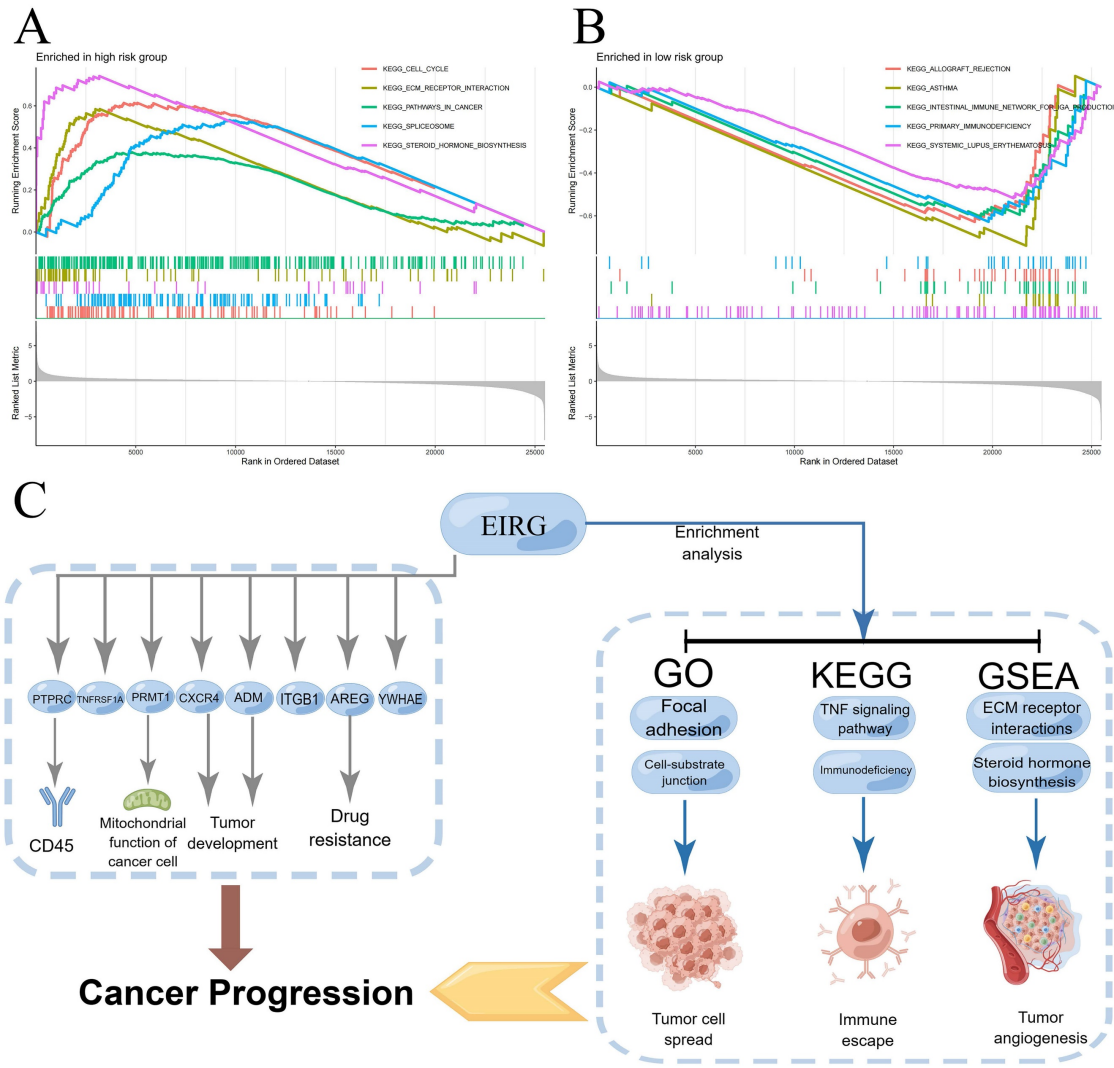
Enrichment analysis, mechanism diagram and tumor mutation burden in different risk groups. Gene set enrichment analysis on the high-risk group and low-risk group (A-B); Mechanism diagram (C); Percentage bar graph showing TMB for different risk subgroups (D-E); Tumor mutation burden in different risk groups (F).

Underlying the mechanism of the signature requires a deeper understanding, and we performed GSEA enrichment analysis. This analysis revealed a notable enrichment of genes related to the production of steroid hormones and the interaction of extracellular matrix receptors within the HG. The LG exhibited gene enrichment primarily related to immunodeficiency and rejection of transplanted organs** (Figure 8A-B)**. The mechanism of the model can be summarized by **Figure 8C**.

### Tumor mutational burden

During this phase, we produced two waterfall charts to obtain a more comprehensive profile of the genetic mutations. The results of our study showed that TP53, TTN, MUC16, CSMD3, and RYR2 had the highest percentage of mutations in both groups, with a greater frequency observed in the HG group **(Figure S8A-B)**. The HG had a significantly greater TMB than did the LG **(Figure S8C)**.

### Tumor immune infiltration status analysis

**Figure 9:**
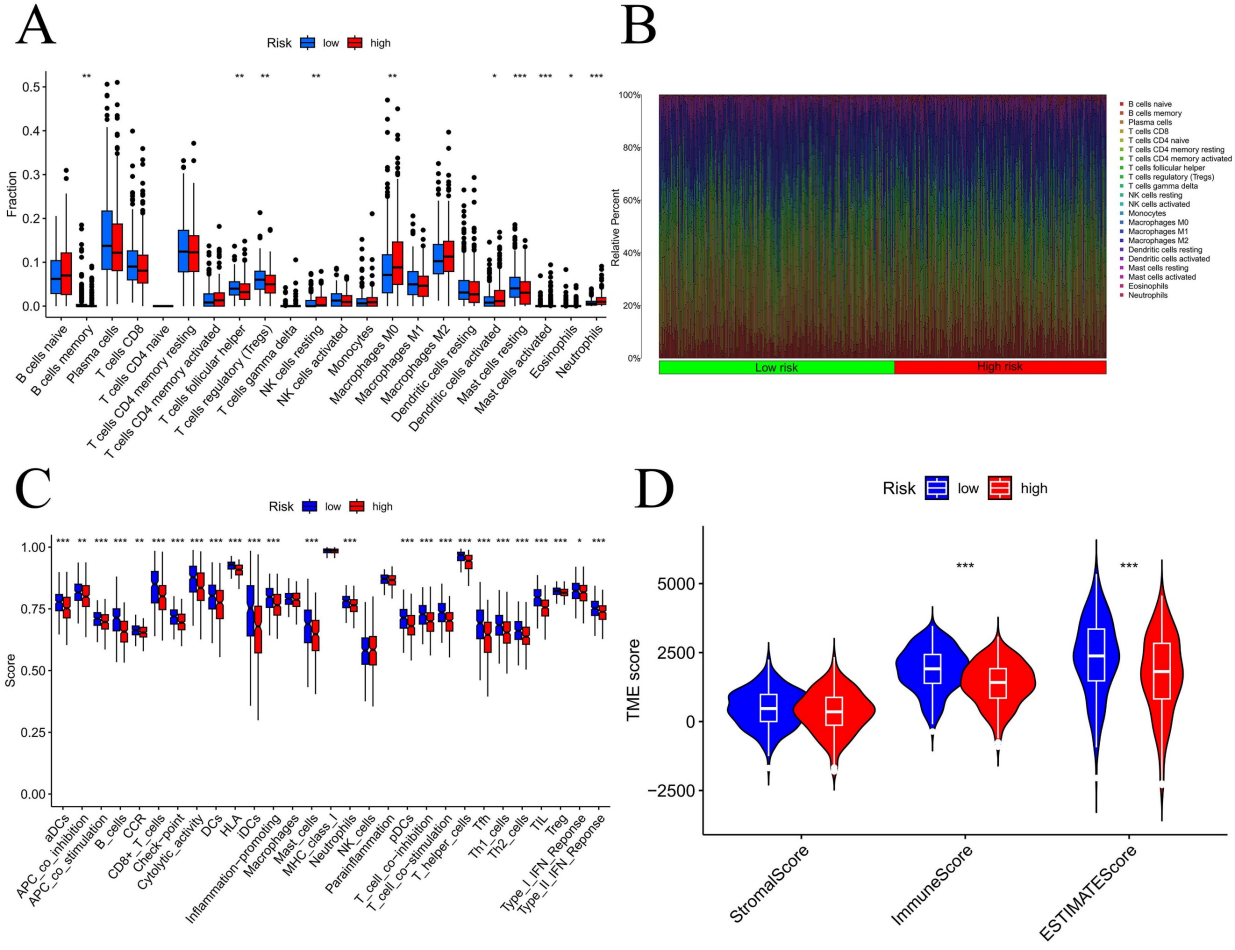
Analysis of tumor immune microenvironment. Difference expression levels of 22 types of tumor-infiltrating immune cells (A-B); immune-related functions between low-risk and high-risk groups (C); Violin plots of differences in stromal scores, immune scores, ESTIMATE scores (D); TIDE algorithm of the high-risk group and low-risk group (E); Differences in risk scores between response and non-response groups (F). *p < 0.05, **p < 0.01, ***p < 0.001, ****p < 0.0001.

We initially examined the link between the risk score and the expression of genes related to immune checkpoints. The findings indicated that as the CD276 expression level increased, the risk score also increased **(Figure S9A)**. Additionally, the outcomes revealed an inverse correlation between the expression of CD27 or BTLA and that of CD40LG **(Figure S9B-D)**. To evaluate the infiltration of immune cells within the TME, we utilized the CIBERSORT function in R software. According to **Figure 9A-B**, the number of activated mast cells and neutrophils were considerably greater in the HG group than in the LG group. Compared with the HG, the LG have more resting mast cells. Most immune-related functional levels were greater in the LG than in the HG **(Figure 9C)**. According to the TME analysis, LG patients had notably greater immune and ESTIMATE scores than did HG patients, but no obvious difference was observed in the stromal score **(Figure 9D)**.

### Immunotherapy response and drug sensitivity analysis

One of our research objectives was to evaluate the response to immunotherapy in the HG and LG through the application of the TIDE algorithm. The TIDE scores were greater in the HG than in the LG **(Figure S10A)**. Furthermore, **Figure S10B** demonstrated a notable increase in the risk score for the nonresponsive group compared to the responsive group. Our study findings revealed a greater likelihood of immune evasion in the HG than in LG, resulting in a diminished effectiveness of immunotherapy. We subsequently performed a drug sensitivity analysis and observed that the IC50 values of ribociclib, SB216763, doramapimod, and BMS754807 were reduced in the LG **(Figure S11A-D)**. Conversely, in HG, SCH772984, BI2536,5-ffluorouracil, and VX11e exhibited lower IC50 values **(Figure S11E-H)**. All drug sensitivity information is contained in **Table S6**.

### *In vitro* experimental validation of the risk models

The immunohistochemical staining images from the HPA database were used to observe the protein expression of each EIRG in LUAD tissues and tissues of normal origin, as shown in **Figure S12A**.

RT‒qPCR **(Figure S12B-C)** revealed that among the EIRGs, PTPRC and CXCR4 were highly expressed in the BEAS-2B cell line. The expression levels of TNFRSF1A, YWHAE, ADM, and AREG were elevated in the H1395 and H1975 cell lines; however, PRMT1 expression was not notably different. Compared to those in normal cell lines, H1975 cells exhibited significantly greater expression of ITGB1, yet no discernible difference in its expression between H1375 cells and healthy cell lines was observed.

## Discussion

The mortality rate and prevalence of lung cancer are notably high 1, and LUAD is a major pathology 2. However, traditional TNM staging is inadequate for predicting the prognosis of lung adenocarcinoma 3. In recent years, the use of biomarkers to construct predictive models for prognosis has been increasing. Consequently, we developed a prognostic model for LUAD using EIRGs and investigated the underlying molecular mechanisms involved. An examination of the eight-gene prognostic model revealed that LUAD patients classified as high risk, with a risk score equal to or exceeding 4.65, experienced a more unfavorable prognosis. The nomograph generated from this model was shown to possess significant predictive power. Analysis of the enrichment data additionally suggested that the main factors impacting HG were the synthesis of steroid hormones and the interaction with ECM receptors. Primary immunodeficiency and allograft rejection were the main areas of gene enrichment in LG patients. The TMB in HGs was greater than that in the LG. According to the findings of the TIDE analysis, the group at greater risk exhibited a greater level of resistance to immunotherapy.

In this study, eight EIRGs were selected as prognostic biomarkers (including TNFRSF1A, CXCR4, YWHAE, PRMT1, ADM, ITGB1, AREG and PTPRC). All of these genes are related to survival in patients with lung adenocarcinoma. CXC chemokine receptors play a significant role in immune surveillance, inflammation, tissue fostering and maintenance 19. A remarkable increase in the survival rate of patients with lung adenocarcinoma was observed due to a notable increase in CXCR4 expression 19. In the protein interaction network, PTPRC was most likely the core gene of the network. The PTPRC gene encodes the protein tyrosine phosphatase CD45 20 and its high expression is linked to a favorable prognosis in patients with LUAD 21. It has been demonstrated that overexpression of YWHAE strengthens the invasiveness of breast cancer cells 22, but no other studies have probed the impact of YWHAE on the outcome of LUAD. Preexisting research has shown that the overexpression of TNFRSF1A, ADM, ITGB1, PRMT1, and AREG is associated with an unfavorable prognosis in patients 23-27. The model based on the above eight EIRGs showed that it has good predictive value, and similar results were obtained in the GEO external cohort. The prognostic model based on the endothelium also possesses strong predictive value when gauging the prognosis of various other types of cancer. Studies have confirmed that endothelial cell-related prognostic indicators have good predictive value for renal clear cell carcinoma 28. Hence, EIRGs can serve as dependable biological indicators for predicting the outcome of patients suffering from LUAD.

Then, we further analyzed the mechanism of the model. We carried out GSEA and found that most of the genes associated with steroid hormone biosynthesis and ECM receptor interactions in the HG. VEGF, VEGF-B, and VEGF-C expression in human breast cancer cells can be stimulated by estrogen and androgens 29. Among the primary cytokines that mediate tumor vascular growth, VEGF plays a key role. We speculate that steroid hormones may have similar effects on lung adenocarcinoma tumors. Disruption of the ECM balance in the TME can strongly affect the development of tumors, angiogenesis, metastasis, immunosuppression, and resistance to drugs 30. Hence, the inhibition of ECM-receptor interactions may present new possibilities for the development of cancer therapies 31. The enrichment pathway results in the LG indicate that individuals at low risk may suffer from immune deficiency, specifically in primary immunodeficiency and allograft rejection. According to the TMB analysis, HG patients exhibited an elevated TMB, with TP53 being the gene most commonly mutated in HG patients. A previous study demonstrated significant correlations between TP53 mutation and resistance to treatment and between TP53 mutation and the outcome of advanced lung cancer 32. This may clarify the reason behind the higher TMB in the HG. The findings indicated that TME in the HG had more activated mast cells and neutrophils, whereas TME in the LG had a greater quantity of resting mast cells. These findings highlight the essential role of mast cells in the TME. Earlier studies have shown that mast cells can generate factors that stimulate the growth of tumor vessels and lymphatics 24 and disperse a variety of proteases that breakdown the cell matrix, thus facilitating the far-reaching spread of tumor cells 33. The group with greater danger exhibited significantly fewer immune functions than did the group with lesser risk in terms of immune-related functions. The immunosuppressive effects of the TME in high-risk patients were demonstrated by these negative correlation findings. The low survival rate observed in the HG may be attributed to dysregulation of the immune system. In summary, we explored the molecular mechanisms involved in the differences among different risk groups and provided possible research directions.

Our analysis of several common immune checkpoint genes revealed that concomitant with the increase in CD276 expression, the risk score increased. An increase in CD276 expression has the potential to enhance the proliferation of cancer cells toward lymph nodes and reduce the lymphocyte count 34. TIDE provided additional insight into the immunotherapy prediction ability of the model, which was confirmed by the findings of the present study, indicating that there is a greater possibility of LG patients benefiting from immunotherapy. These findings also confirmed that our model can predict treatment response. Drug sensitivity tests were also conducted for different risk groups. Due to the unfavorable outlook in the HG, our primary focus was on examining the responsive medications within the cohort. Ribociclib, a cyclin-dependent kinase (CDK) 4/6 inhibitor, has received authorization for the treatment of HR+ and HER2- breast cancer patients 35, and it is revealed to be one of the most sensitive medications for the HG. Interestingly, our enrichment analysis revealed that the group at high risk was enriched in the pathway associated with the biosynthesis of steroid hormones, this may be the reason why patients in the HG are sensitive to ribociclib. Patients diagnosed with LUAD at varying risk levels can utilize these findings to select personalized treatment options that are suitable for their specific needs.

Sc-RNAseq is a powerful technique that can also address cell heterogeneity that conventional transcriptome sequencing cannot. Bulk RNA sequencing data can be used to verify the accuracy of sc-RNAseq, thus improving the reliability of the results of this study. Utilizing sc-RNAseq technology, our study reveals the diversity of gene expression at the cellular level, which makes individualized precision treatment possible. RT‒qPCR was used to verify the stability of the model. We explored differences in signaling pathways and the TME between patients with different risk levels through multiple research methods. Nonetheless, this investigation's potential constraint lies in its reliance on previously existing databases, as it was retrospective. Hence, future predictive studies encompassing broader samples are essential for its validation.

## Conclusion

The risk score and nomogram from this study hold outstanding predictive value for individuals suffering from LUAD. In addition, our research revealed that the synthesis of steroid hormones and their interaction with extracellular matrix receptors might play crucial roles in the HG. This research could impact the development of novel treatment methods for LUAD, but further experimental and clinical validation are needed.

## Supplementary Material

Supplementary figures and tables.

## Figures and Tables

**Table 1:** Clinical information of the patients in the test and train groups.

Characteristics	Train cohort (n=522)			Test cohort (n=443)	
	n	%		n	%
**Age**					
<65	262	50.19		231	52.14
>65	241	46.17		212	47.86
Unknown	19	3.64			
**Status**					
Alive	334	63.98		207	46.73
Dead	188	36.02		236	53.27
**Gender**					
Female	280	54.02		220	49.66
Male	242	45.98		223	50.34
**Stage**					
Stage I	249	47.70			
Stage II	124	23.75			
Stage III	85	16.29			
Stage IV	26	4.98			
Unknown	8	1.53			
**T stage**					
T1	172	32.95		150	33.86
T2	281	53.83		251	56.66
T3	47	9.00		28	6.32
T4	19	3.65		12	2.71
Unknown	3	0.57		2	0.45
**M stage**					
M0	353	67.62			
M1	25	4.79			
Unknown	144	27.59			
**N stage**					
N0	335	64.18		299	67.49
N1	98	18.77		88	19.87
N2	75	14.37		53	11.96
N3	2	0.38			
Unknown	12	2.30		3	0.68

**Abbreviations:** T stage: Tumor stage; N stage**:** Node stage; M stage**:** metastasis stage.

**Table 2:** Clinical information for 507 patients in different risk categories.

Characteristics	High-risk group (n=253)			Low-risk group (n=254)	
	n	%		n	%
**Age**					
<65	129	50.79		110	43.31
>65	124	49.21		140	55.12
Unknown				4	1.57
**Status**					
Alive	140	55.34		184	72.44
Dead	113	44.66		70	27.56
**Gender**					
Female	117	46.25		154	60.63
Male	136	53.75		100	39.37
**Stage**					
Stage I	102	40.32		169	66.54
Stage II	69	27.27		50	19.69
Stage III	58	22.92		25	9.84
Stage IV	19	7.51		7	2.75
Unknown	5	1.98		3	1.18
**T stage**					
T1	58	22.92		111	43.70
T2	149	58.89		122	48.03
T3	30	11.86		15	5.91
T4	15	5.93		4	1.57
Unknown	1	0.40		2	0.79
**M stage**					
M0	175	69.17		163	64.17
M1	19	7.51		6	2.37
Unknown	59	23.32		85	33.46
**N stage**					
N0	139	54.94		187	73.62
N1	60	23.71		34	13.39
N2	51	20.16		22	8.66
N3	1	0.40		1	0.39
Unknown	2	0.79		10	3.94

**Abbreviations:** T stage: Tumor stage; N stage**:** Node stage; M stage**:** metastasis stage.

## Abbreviations

EIRG: Endothelial cell immune-related gene
GEO: the gene expression omnibus
GO: Gene Ontology
GSEA: Gene set enrichment analysis
HG: high-risk group
KEGG: Kyoto Encyclopedia of Genes and Genomes
LASSO: Least absolute shrinkage and selection operator
LG: low-risk group
LUAD: Lung adenocarcinoma
OS: Overall survival
PCA: Principal component analysis
ROC: receiver operating characteristic
sc-RNAseq: single-cell RNA sequencing
TCGA: The Cancer Genome Atlas
TMB: Tumor Mutation Burden
TME: tumor microenvironment

## References

[1] Sung H, Ferlay J, Siegel RL, Laversanne M, Soerjomataram I, Jemal A (2021). Global Cancer Statistics 2020: GLOBOCAN Estimates of Incidence and Mortality Worldwide for 36 Cancers in 185 Countries. CA Cancer J Clin.

[2] Siegel RL, Miller KD, Wagle NS, Jemal A (2023). Cancer statistics, 2023. CA Cancer J Clin.

[3] Motzer RJ, Jonasch E, Agarwal N, Alva A, Baine M, Beckermann K (2022). Kidney Cancer, Version 3.2022, NCCN Clinical Practice Guidelines in Oncology. J Natl Compr Canc Netw.

[4] Shi R, Bao X, Unger K, Sun J, Lu S, Manapov F (2021). Identification and validation of hypoxia-derived gene signatures to predict clinical outcomes and therapeutic responses in stage I lung adenocarcinoma patients. Theranostics.

[5] Hinshaw DC, Shevde LA (2019). The Tumor Microenvironment Innately Modulates Cancer Progression. Cancer Res.

[6] Zhao S, Ji W, Shen Y, Fan Y, Huang H, Huang J (2022). Expression of hub genes of endothelial cells in glioblastoma-A prognostic model for GBM patients integrating single-cell RNA sequencing and bulk RNA sequencing. BMC Cancer.

[7] Zhang J, Liu X, Huang Z, Wu C, Zhang F, Han A (2023). T cell-related prognostic risk model and tumor immune environment modulation in lung adenocarcinoma based on single-cell and bulk RNA sequencing. Comput Biol Med.

[8] Stuart T, Butler A, Hoffman P, Hafemeister C, Papalexi E, Mauck WM 3rd (2019). Comprehensive Integration of Single-Cell Data. Cell.

[9] Do VH, Canzar S (2021). A generalization of t-SNE and UMAP to single-cell multimodal omics. Genome Biol.

[10] Aran D, Looney AP, Liu L, Wu E, Fong V, Hsu A (2019). Reference-based analysis of lung single-cell sequencing reveals a transitional profibrotic macrophage. Nat Immunol.

[11] Gene Ontology Consortium (2015). going forward. Nucleic Acids Res.

[12] Kanehisa M, Furumichi M, Tanabe M, Sato Y, Morishima K (2017). KEGG: new perspectives on genomes, pathways, diseases and drugs. Nucleic Acids Res.

[13] Tibshirani R (1997). The lasso method for variable selection in the Cox model. Stat Med.

[14] Chen Q, Wang S, Lang J-H (2021). Development and validation of nomogram with tumor microenvironment-related genes and clinical factors for predicting overall survival of endometrial cancer. J Cancer.

[15] Subramanian A, Tamayo P, Mootha VK, Mukherjee S, Ebert BL, Gillette MA (2005). Gene set enrichment analysis: a knowledge-based approach for interpreting genome-wide expression profiles. Proc Natl Acad Sci U S A.

[16] Newman AM, Liu CL, Green MR, Gentles AJ, Feng W, Xu Y (2015). Robust enumeration of cell subsets from tissue expression profiles. Nat Methods.

[17] Bindea G, Mlecnik B, Tosolini M, Kirilovsky A, Waldner M, Obenauf AC (2013). Spatiotemporal dynamics of intratumoral immune cells reveal the immune landscape in human cancer. Immunity.

[18] Jiang P, Gu S, Pan D, Fu J, Sahu A, Hu X (2018). Signatures of T cell dysfunction and exclusion predict cancer immunotherapy response. Nat Med.

[19] Hu L-T, Deng W-J, Chu Z-S, Sun L, Zhang C-B, Lu S-Z (2022). Comprehensive analysis of CXCR family members in lung adenocarcinoma with prognostic values. BMC Pulm Med.

[20] Porcu M, Kleppe M, Gianfelici V, Geerdens E, De Keersmaecker K, Tartaglia M (2012). Mutation of the receptor tyrosine phosphatase PTPRC (CD45) in T-cell acute lymphoblastic leukemia. Blood.

[21] Wei J, Fang D, Zhou W (2021). CCR2 and PTPRC are regulators of tumor microenvironment and potential prognostic biomarkers of lung adenocarcinoma. Ann Transl Med.

[22] Yang Y-F, Lee Y-C, Wang Y-Y, Wang C-H, Hou M-F, Yuan S-SF (2019). YWHAE promotes proliferation, metastasis, and chemoresistance in breast cancer cells. Kaohsiung J Med Sci.

[23] Zhang X, Ye T, Li M, Yan H, Lin H, Lu H (2022). Association of Polymorphisms in Inflammation Genes With the Prognosis of Advanced Non-Small Cell Lung Cancer Patients Receiving Epidermal Growth Factor Receptor Tyrosine Kinase Inhibitors. Front Oncol.

[24] Kang YJ, Bang B-R, Han KH, Hong L, Shim E-J, Ma J (2015). Regulation of NKT cell-mediated immune responses to tumours and liver inflammation by mitochondrial PGAM5-Drp1 signalling. Nat Commun.

[25] Wu J, Wang W, Li Z, Ye X (2022). The prognostic and immune infiltration role of ITGB superfamily members in non-small cell lung cancer. Am J Transl Res.

[26] Madreiter-Sokolowski CT, Győrffy B, Klec C, Sokolowski AA, Rost R, Waldeck-Weiermair M (2017). UCP2 and PRMT1 are key prognostic markers for lung carcinoma patients. Oncotarget.

[27] Busser B, Coll JL, Hurbin A (2009). The increasing role of amphiregulin in non-small cell lung cancer. Pathol Biol (Paris).

[28] Li D-X, Yu Q-X, Zeng C-X, Ye L-X, Guo Y-Q, Liu J-F (2023). A novel endothelial-related prognostic index by integrating single-cell and bulk RNA sequencing data for patients with kidney renal clear cell carcinoma. Front Genet.

[29] Ruohola JK, Valve EM, Karkkainen MJ, Joukov V, Alitalo K, Härkönen PL (1999). Vascular endothelial growth factors are differentially regulated by steroid hormones and antiestrogens in breast cancer cells. Mol Cell Endocrinol.

[30] Zeltz C, Primac I, Erusappan P, Alam J, Noel A, Gullberg D (2020). Cancer-associated fibroblasts in desmoplastic tumors: emerging role of integrins. Semin Cancer Biol.

[31] Dzobo K, Senthebane DA, Dandara C (2023). The Tumor Microenvironment in Tumorigenesis and Therapy Resistance Revisited. Cancers (Basel).

[32] Steels E, Paesmans M, Berghmans T, Branle F, Lemaitre F, Mascaux C (2001). Role of p53 as a prognostic factor for survival in lung cancer: a systematic review of the literature with a meta-analysis. Eur Respir J.

[33] Baram D, Vaday GG, Salamon P, Drucker I, Hershkoviz R, Mekori YA (2001). Human mast cells release metalloproteinase-9 on contact with activated T cells: juxtacrine regulation by TNF-alpha. J Immunol.

[34] Picarda E, Ohaegbulam KC, Zang X (2016). Molecular Pathways: Targeting B7-H3 (CD276) for Human Cancer Immunotherapy. Clin Cancer Res.

[35] Braal CL, Jongbloed EM, Wilting SM, Mathijssen RHJ, Koolen SLW, Jager A (2021). Inhibiting CDK4/6 in Breast Cancer with Palbociclib, Ribociclib, and Abemaciclib: Similarities and Differences. Drugs.

