# Supplementary material for: Identification Of Endothelial Cell Immune-related Gene Signature for Lung Adenocarcinoma by Integrated Analysis of Single-cell and Bulk RNA Sequencing Data
Source: J Cancer. 2024 May 20;15(12):3766–80. doi: 10.7150/jca.94501 (PMC11190765; doi:10.7150/jca.94501)
Supplement: Supplementary file 1 — Supplementary figures and tables. [file jcav15p3766s1.pdf]

Supplementary Data

Figure S1 The P-value of each PC and the amount of gene expression in each cluster. 20 PCs were identified based on P-value< 0.05 (A); The heatmap showed the relative expression of genes in 11 clusters (B).

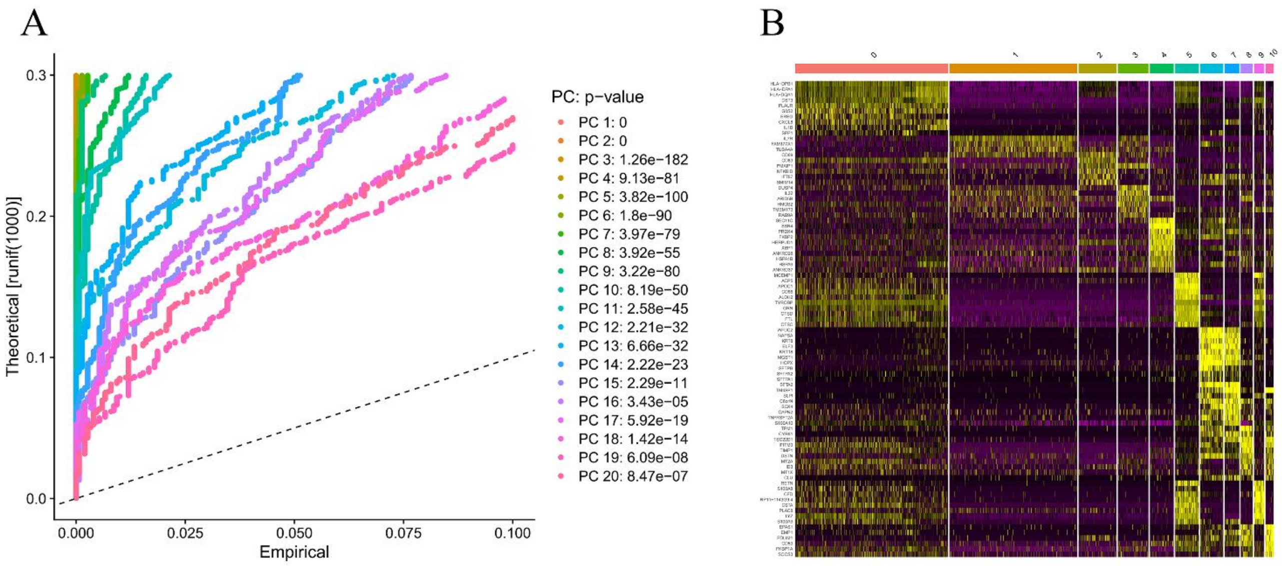

Figure S2 GO enrichment analysis. Circle chart (A); Bar chart (B); Bubble (C).

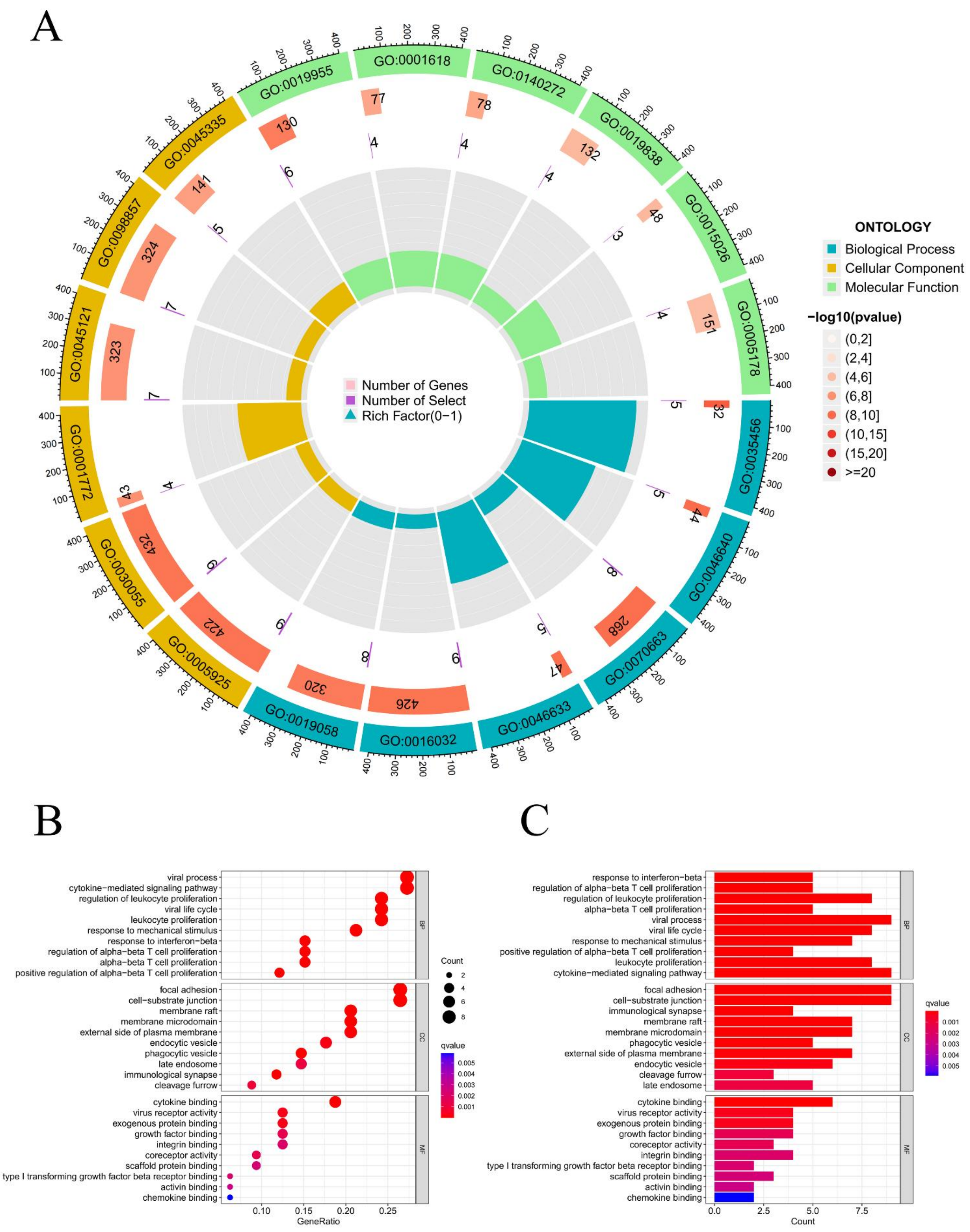

Figure S3 KEGG enrichment analysis. Bubble chart (A); Bar (B).

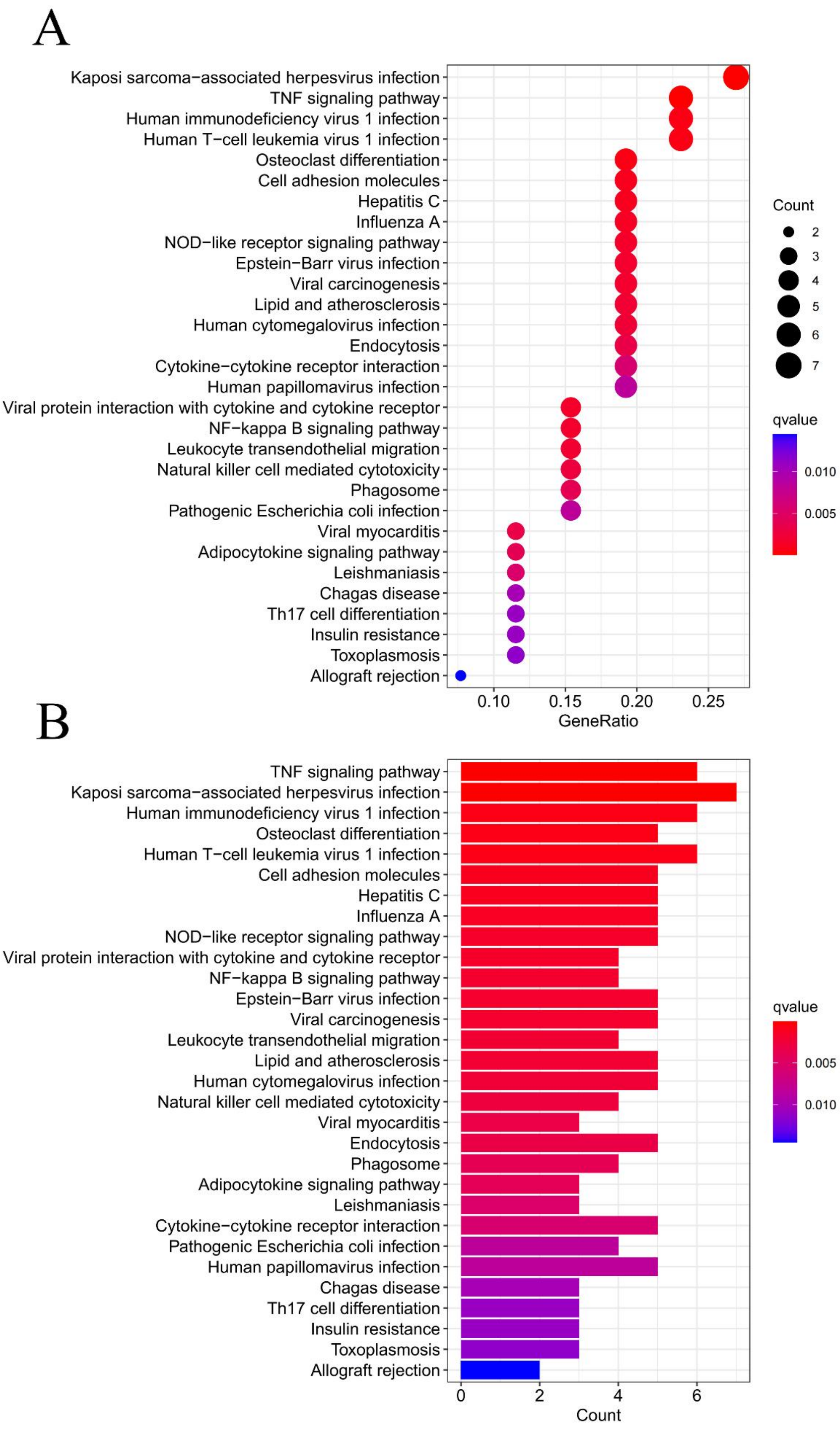

Figure S4 Protein-protein interaction. PPI map (A); Number of adjacent nodes (B).

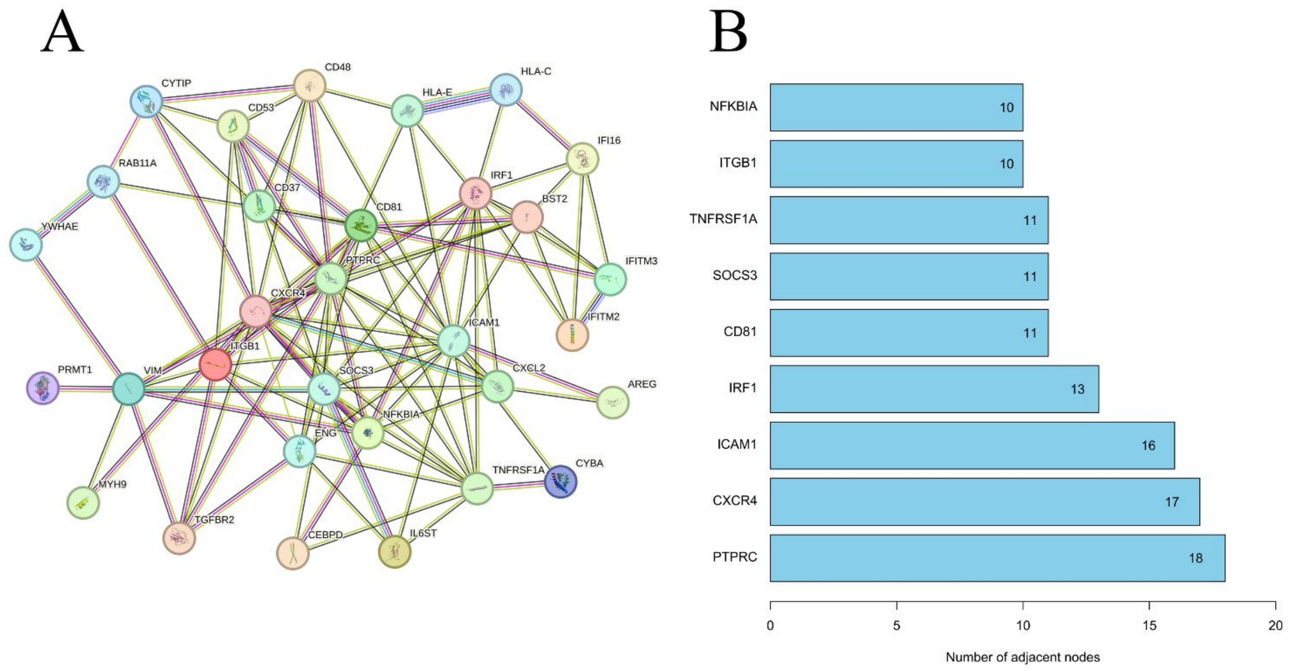

Figure S5 Survival analysis in LUAD patients based on 8 EIRG. Kaplan – Meier curves of each EIRG (A-H).

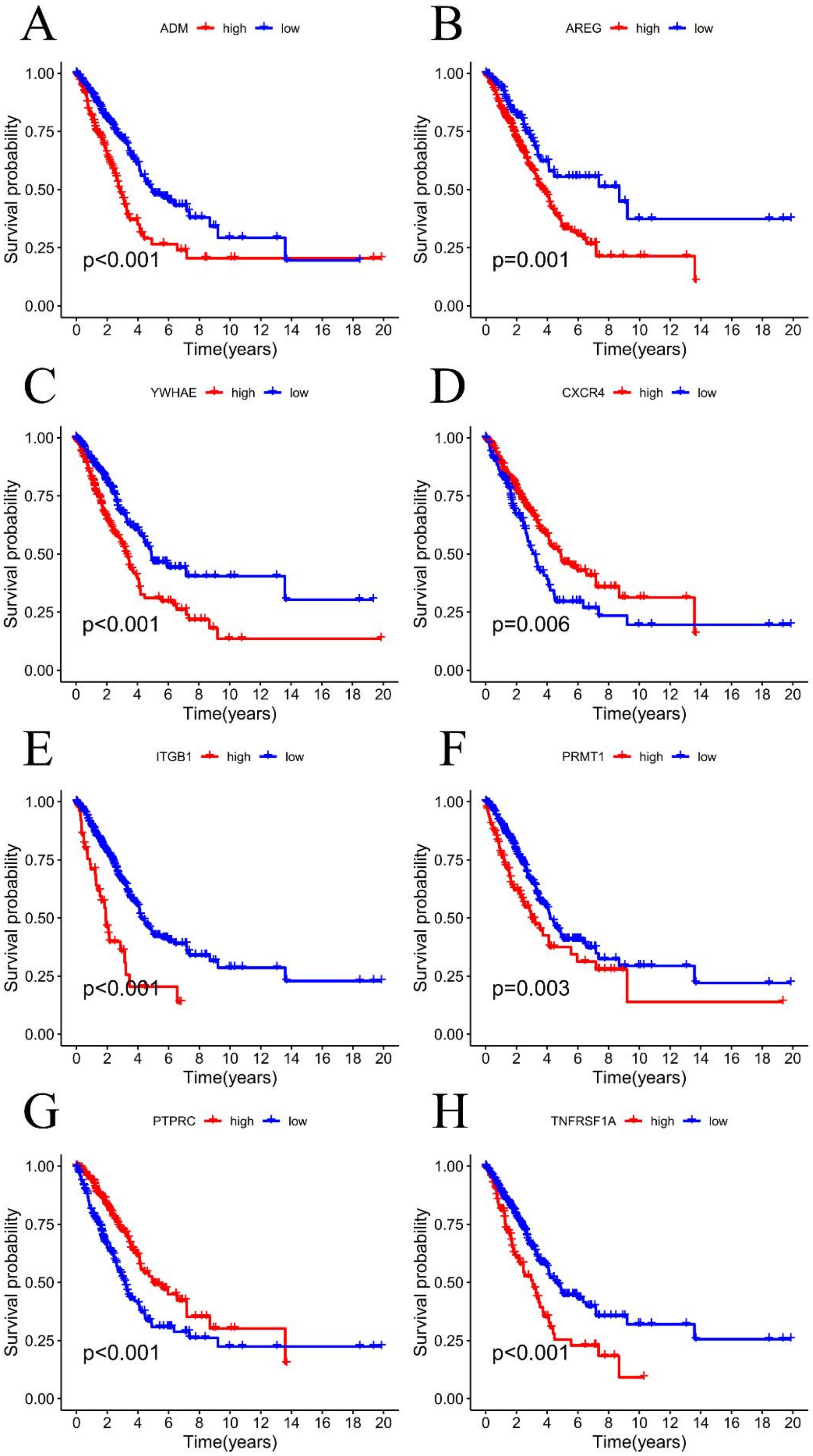

Figure S6 The model prediction effect is validated by the train group and test group. Heat map of 8 endothelial cell immune-related genes expressions (A, B); Risk curve for risk scores (C, D); Scatterplot for the survival status of each patient (E, F).

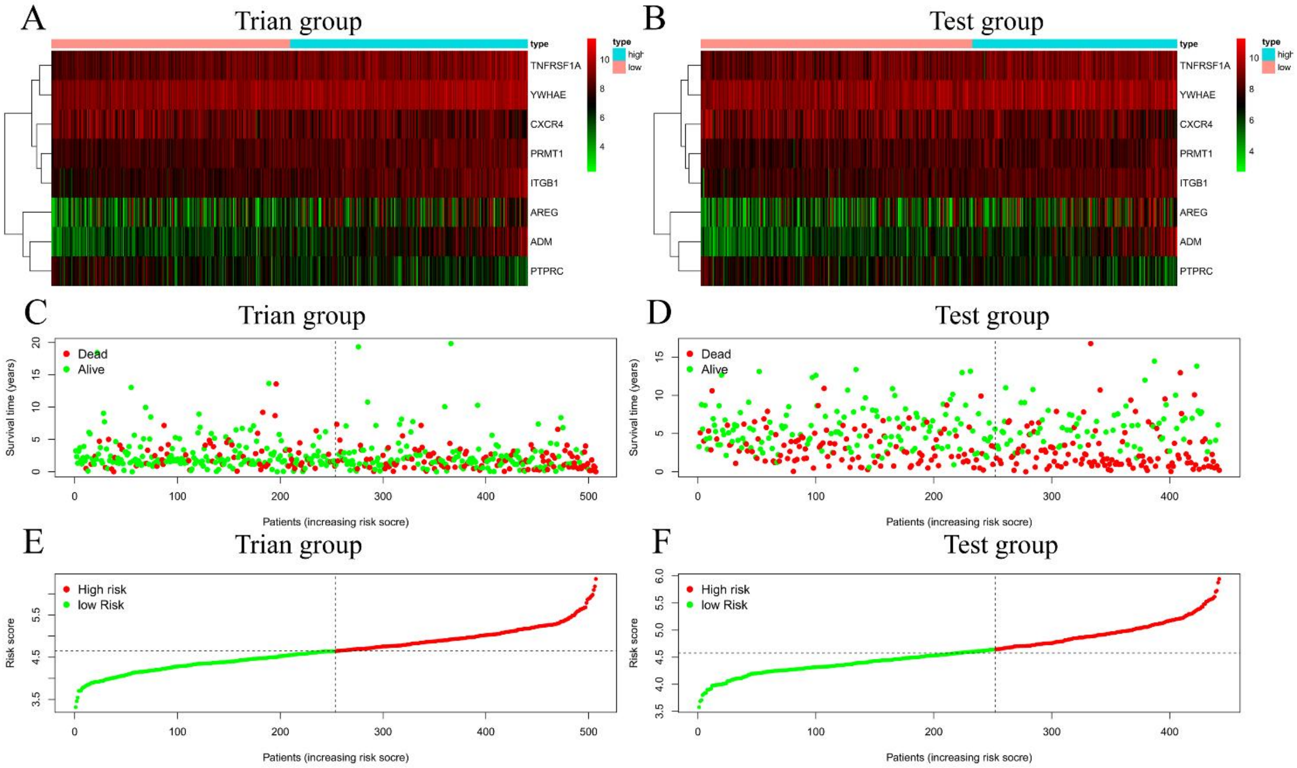

Figure S7 Clinical relevance analysis of different risk groups. Circle plot of clinical relevance (A); Heatmap of clinical relevance (B).

A

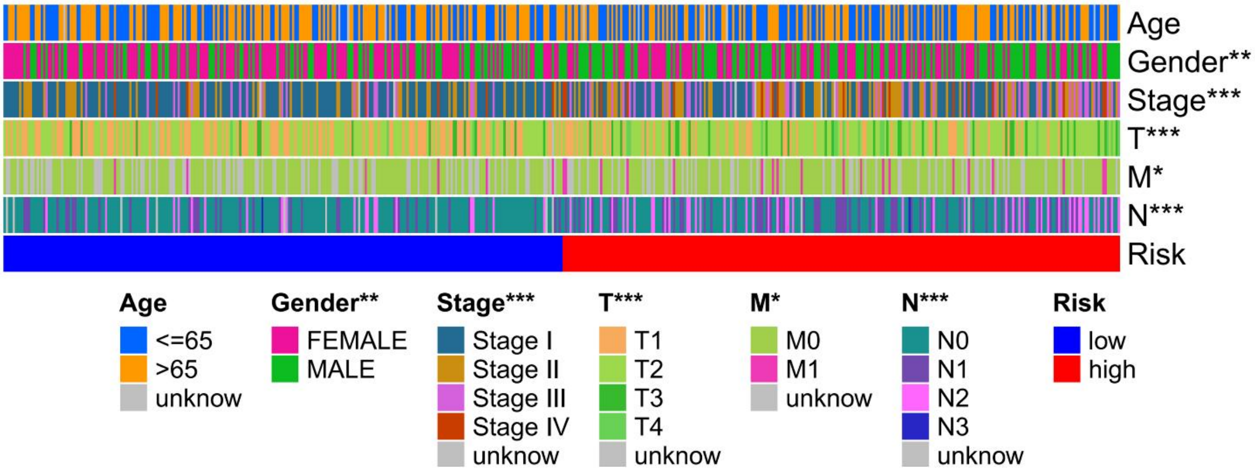

B

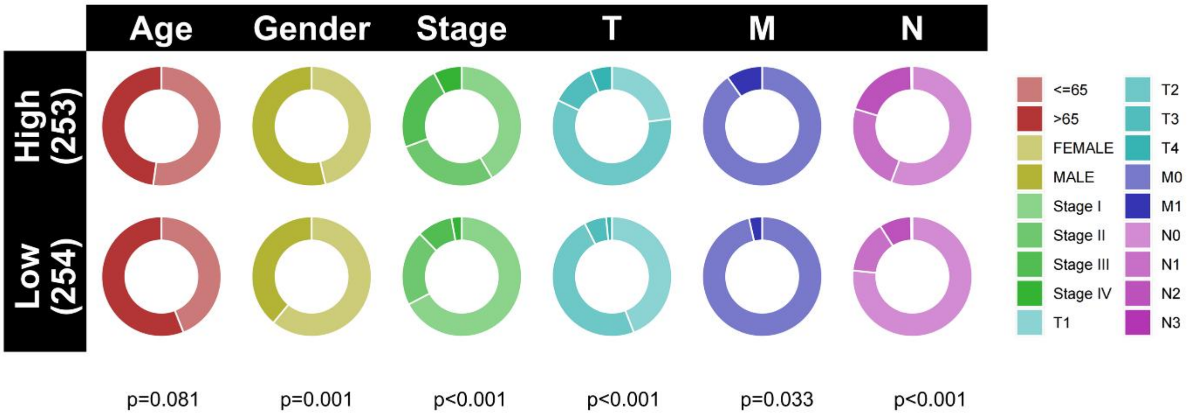

Figure S8 Tumor mutation burden in different risk groups. Percentage bar graph showing TMB for different risk subgroups (A-B); Tumor mutation burden in different risk groups (C).

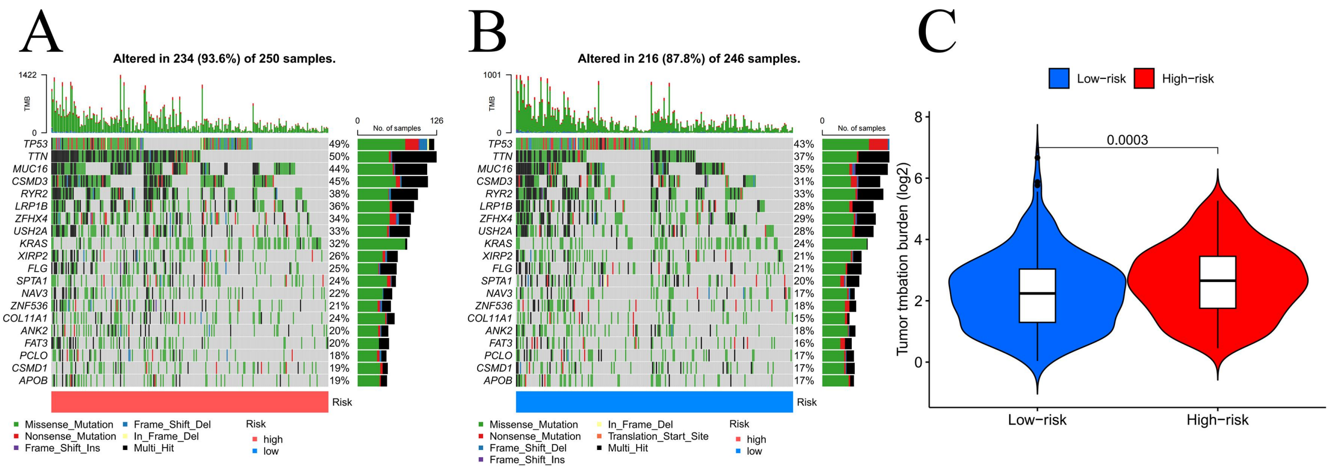

Figure S9 Tumor immune dysfunction and exclusion. TIDE algorithm of the high-risk group and low-risk group (A); Differences in risk scores between response and non-response groups (B).

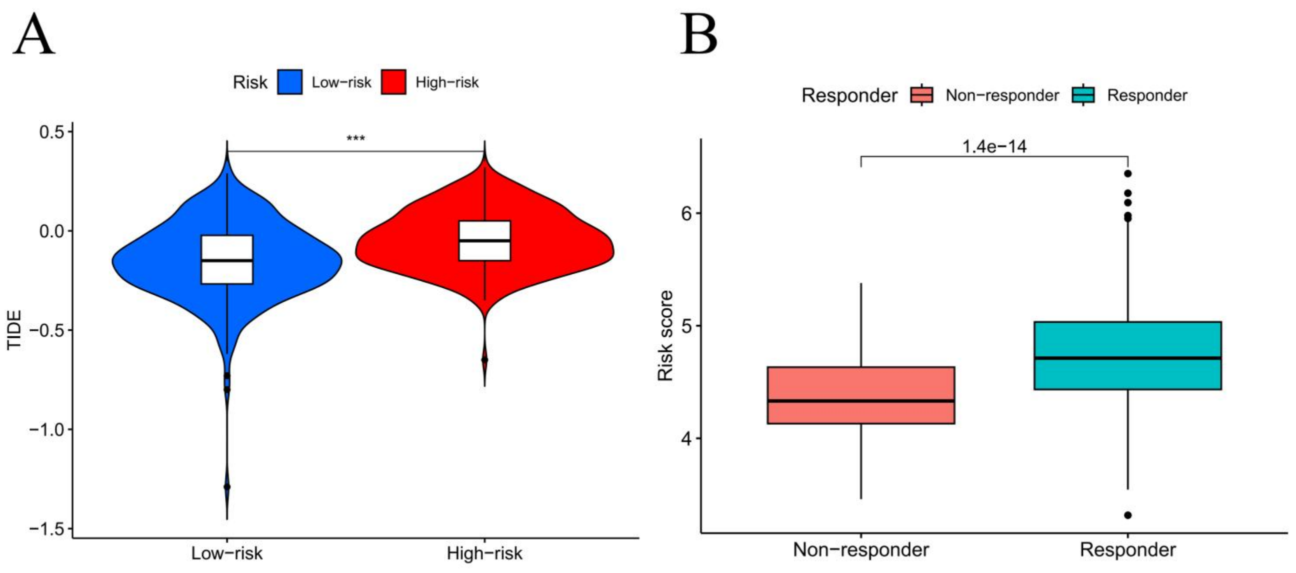

Figure S10 Immune checkpoint analysis. Scatter plot of the correlation between risk scores and immune checkpoint genes (A-D).

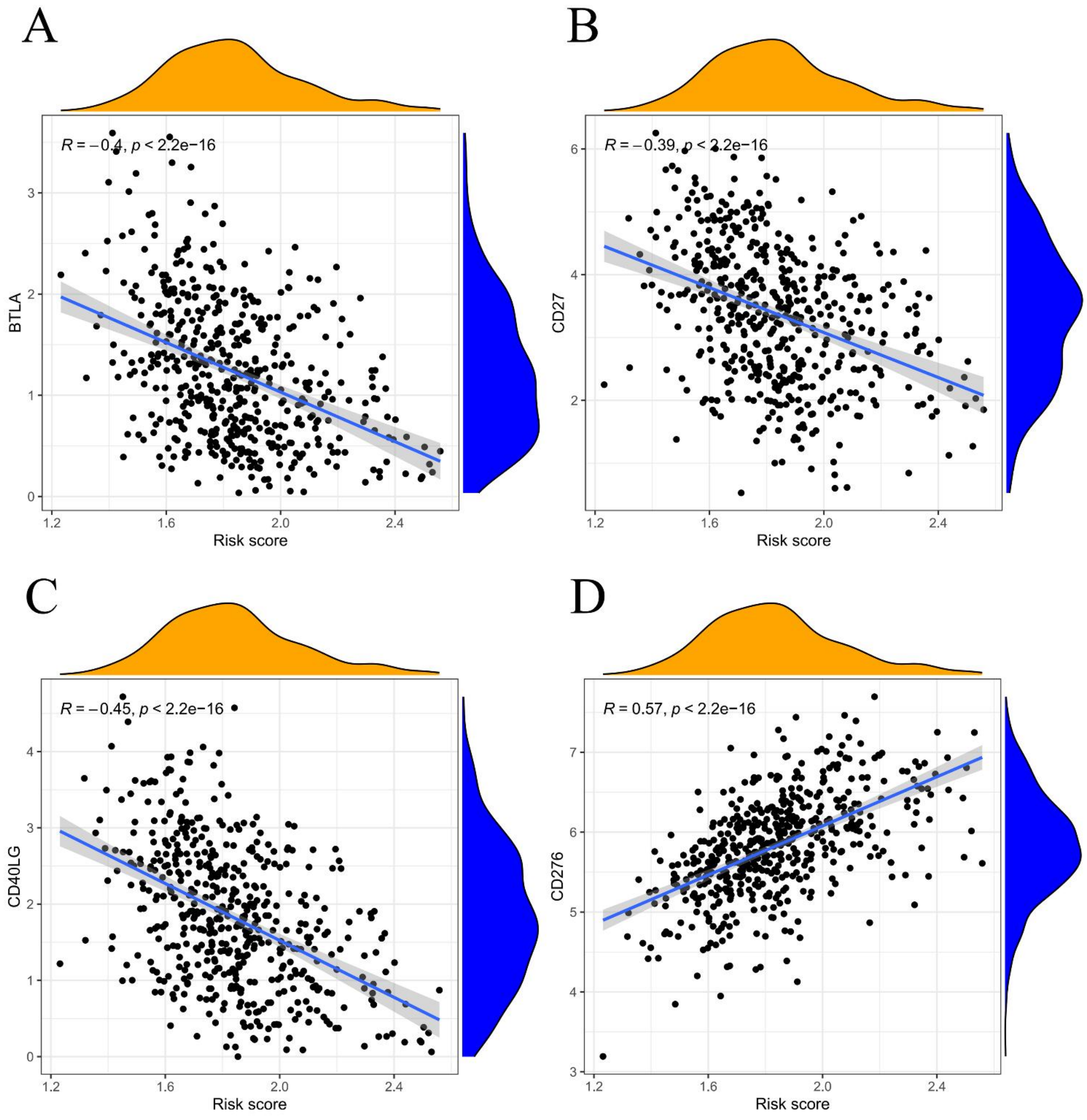

Figure S11 The comparisons in IC50 value of drugs. Ribociclib (A); SB216763 (B); Doramapimod (C); Doramapimod (D); SCH772984 (E); SCH772984 (F); 5-fluorouracil (G); VX (H).

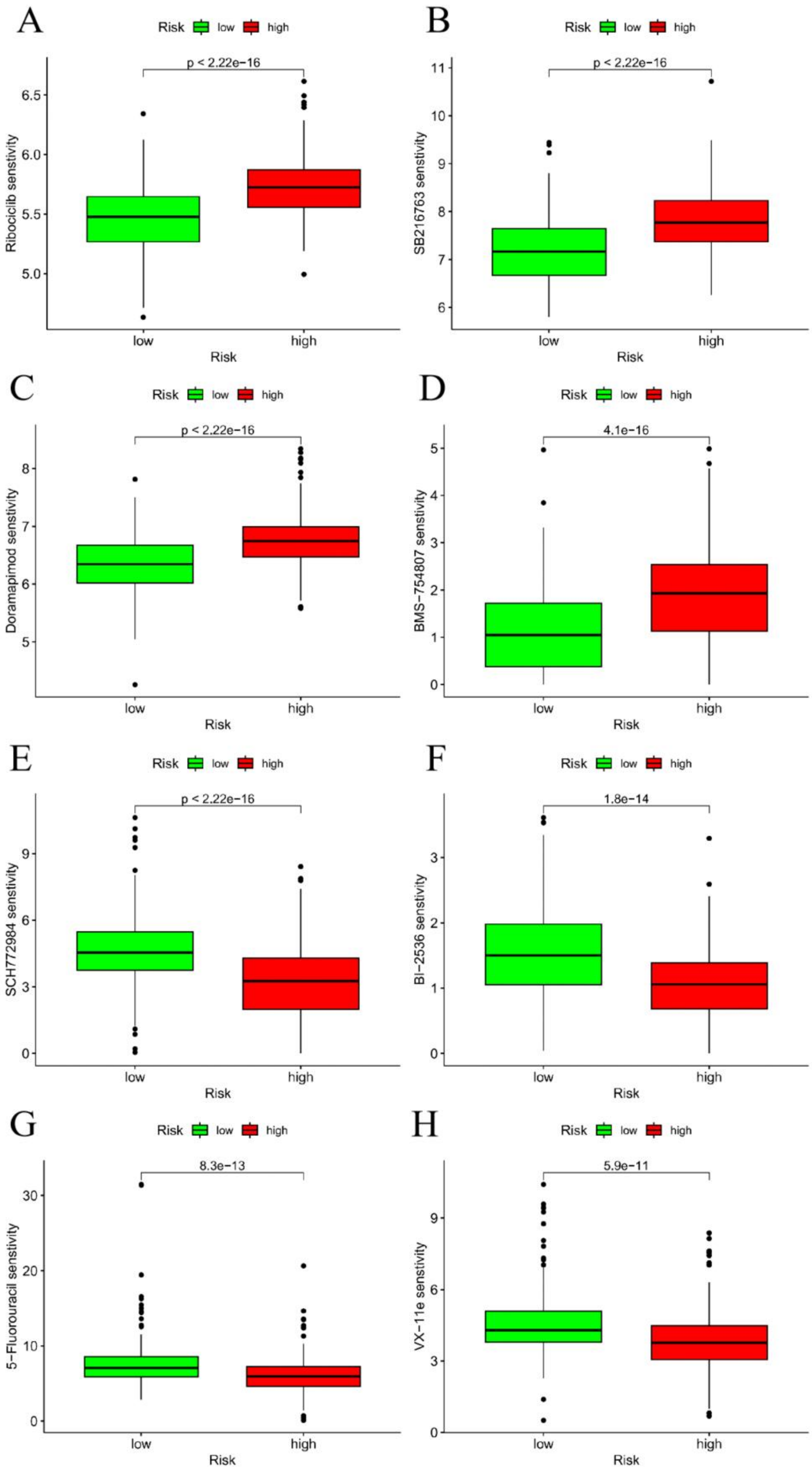

Figure S12 In vitro experimental validation of the risk model. Immunohistochemical staining images of partial EIRGs proteins in LUAD tissue and normal tissue (A); Relative expression of 8 EIRGs in different cell lines (B-C). *p < 0.05, **p < 0.01, ***p < 0.001.

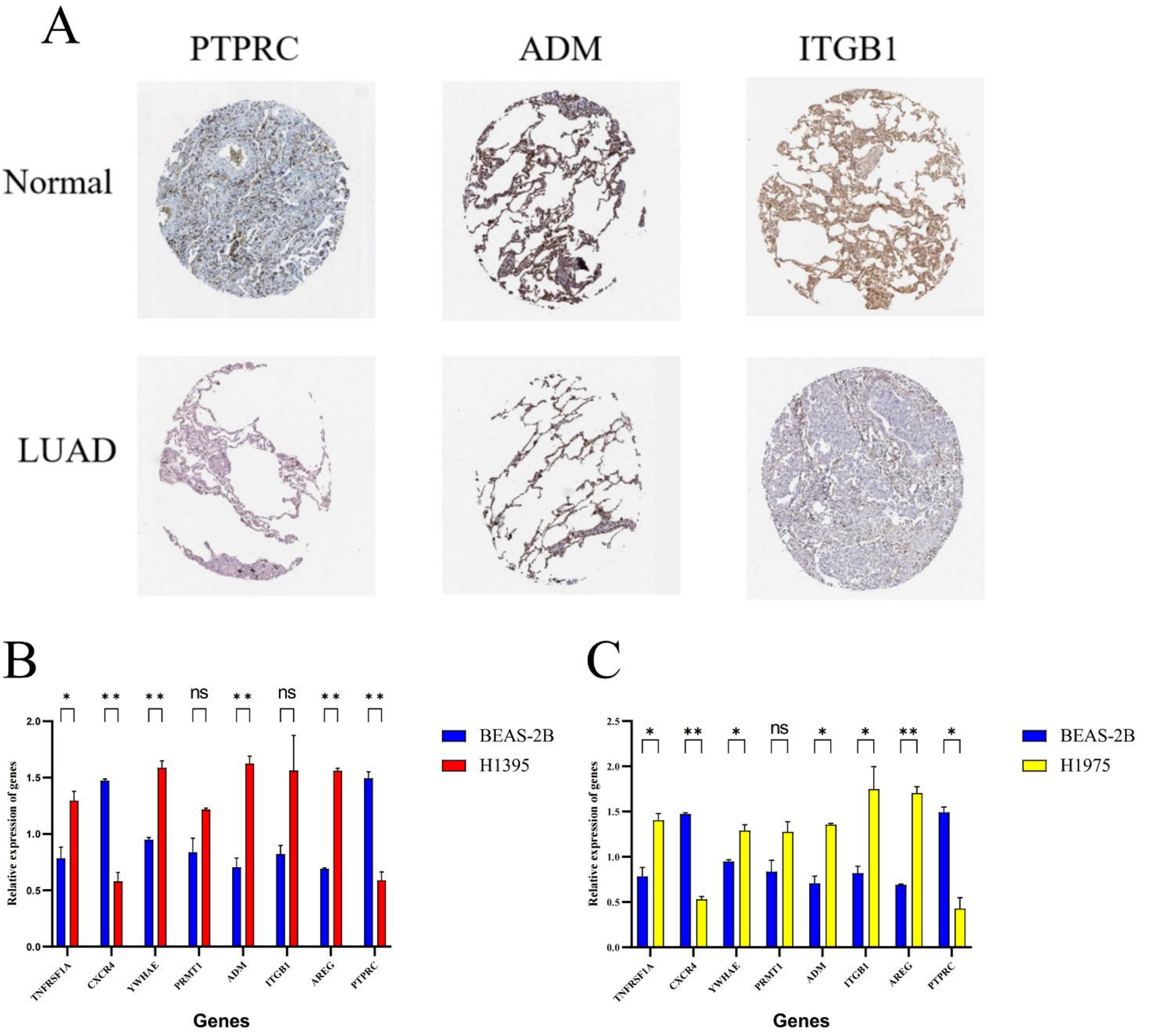

Table S1 Primer sequences for 8 endothelial cell immune-related genes.

Ensembl Gene Id	Primer F	Primer R
ENSG00000067182	TCACCGCTTCAGAAAACCACC	GGTCCACTGTGCAAGAAGAGA
ENSG00000121966	ACTACACCGAGGAAATGGGCT	CCCACAATGCCAGTTAAGAAGA
ENSG00000108953	GATTCGGGAATATCGGCAAATGG	GCTGGAATGAGGTGTTTGTCC
ENSG00000126457	CTTTGACTCCTACGCACACTT	GTGCCGGTTATGAAACATGGA
ENSG00000148926	ATGAAGCTGGTTTCCGTCG	GACATCCGCAGTTCCCTCTT
ENSG00000150093	CCTACTTCTGCACGATGTGATG	CCTTTGCTACGGTTGGTTACATT
ENSG00000109321	GTGGTGCTGTCGCTCTTGATA	CCCCAGAAAATGGTTCACGCT
ENSG00000081237	ACCACAAGTTTACTAACGCAAGT	TTTGAGGGGGATTCCAGGTAAT

Table S2 Endothelial cell immune-related genes from cell.

Gene	log2FC	Percentage (%)	Adjusted p-value
CCL21	3.48	16.8	0
CLDN5	3.15	90.5	0
IGFBP7	2.84	83.8	0
GNG11	2.83	93.3	0
RAMP2	2.8	92.9	0
FCN3	2.71	36.3	0
SPARCL1	2.7	76.4	0
CAV1	2.58	88.4	0
EPAS1	2.45	81.1	0
VWF	2.39	69	0
TNFSF10	2.35	77.4	0
CLEC14A	2.35	73.2	0
IFI27	2.26	87	0
TM4SF1	2.22	89.7	0
SPARC	2.2	72.7	0
EGFL7	2.17	86.1	0
ACKR1	2.08	27.2	0
ID1	2.04	72	0
RAMP3	2.01	62.5	0
TMEM100	2	40.7	0
IFITM3	1.99	97.5	0
ADIRF	1.98	62.2	0
PTRF	1.98	83.7	0
MGP	1.94	72	0
ECSCR	1.89	76.3	0
SDPR	1.85	67.9	0
IGFBP4	1.85	81.5	0
ID3	1.85	75.3	0
AQP1	1.84	57.9	0
TIMP3	1.83	75.3	0
PECAM1	1.82	81.7	0
CTNNAL1	1.8	53.9	0
A2M	1.78	75	0
SLC9A3R2	1.73	65.2	0
CLU	1.71	45.7	0
ARHGAP29	1.7	69.7	0
TFPI	1.66	64	0
HYAL2	1.65	66.1	0
ESAM	1.65	62	0
GPX3	1.64	54.8	0
ENG	1.62	69.5	0
ENPP2	1.58	30	0

SOCS3	1.57	71.1	0
TCF4	1.54	71.5	0
APP	1.54	79	0
CRIP2	1.52	74.5	0
CALCRL	1.49	56.6	0
DNASE1L3	1.45	25.3	0
CD59	1.45	81.8	0
CAV2	1.45	65.1	0
PTPRB	1.44	54.7	0
CD93	1.44	55.1	0
LDB2	1.43	60.7	0
CALD1	1.42	72.4	0
FKBP1A	1.41	89.4	0
RDX	1.41	66.5	0
SPTBN1	1.39	71	0
CCL2	1.38	39.8	0
HES1	1.38	53.6	0
VAMP5	1.38	82.6	0
PCAT19	1.37	58	0
BCAM	1.36	64.4	0
RNASE1	1.36	73.6	0
EMCN	1.35	51.5	0
PLVAP	1.35	40.8	0
COL4A1	1.35	44.9	0
PRSS23	1.34	52.9	0
HLA-E	1.34	97.9	0
NPDC1	1.33	59.4	0
CA4	1.32	24.8	0
PCDH17	1.31	44.6	0
CDH5	1.3	54.8	0
BST2	1.3	74.2	0
HSPG2	1.29	44.6	0
EDN1	1.28	28.8	0
PDLIM1	1.26	70	0
NFIB	1.25	60.4	0
TSC22D1	1.24	55.5	0
IL33	1.22	40	0
ACVRL1	1.21	54.4	0
TSPAN7	1.21	49.6	0
ITM2B	1.21	97	0
NNMT	1.18	44.6	0
POSTN	1.17	30.9	0
ICAM2	1.16	59.5	0
EMP2	1.16	67	0
CNN3	1.16	56.2	0
S100A16	1.15	68.6	0

CCDC85B	1.15	76.6	0
SH3BP5	1.14	60.6	0
SERPINB6	1.13	64.7	0
KLF4	1.12	58.6	0
ROBO4	1.12	47.3	0
COL4A2	1.12	40.1	0
SNCG	1.12	40.7	0
EDNRB	1.11	24.8	0
MARCKSL1	1.11	72.2	0
MGST2	1.1	61.3	0
EMP1	1.09	45.6	0
PALMD	1.08	40.6	0
ACE	1.07	37.5	0
LIFR	1.07	38	0
ADGRL4	1.06	41.1	0
CD34	1.06	42.7	0
TGM2	1.05	53.6	0
C10orf10	1.04	28.5	0
JAM2	1.04	40.6	0
NRN1	1.04	46.7	0
NOTCH4	1.04	37.6	0
YBX3	1.04	62.8	0
SLCO2A1	1.03	37.6	0
NOSTRIN	1.03	38.2	0
ADGRF5	1.02	43.9	0
ETS2	1.01	56.5	0
RHOC	1.01	73.3	0
TIE1	0.99	44.4	0
FAM107A	0.98	32	0
GBP4	0.98	40.4	0
CTGF	0.98	28.3	0
RAB11A	0.98	68.6	0
STOM	0.98	64.8	0
ITM2A	0.97	55.7	0
THBD	0.97	42.6	0
LYVE1	0.97	27.8	0
FXYD6	0.96	41.1	0
SLC6A4	0.96	16.8	0
FAM167B	0.95	32.6	0
PODXL	0.93	36.8	0
PRCP	0.93	42.9	0
EFNB2	0.93	33.7	0
STXBP6	0.93	35.1	0
TM4SF18	0.92	35	0
FLT1	0.92	34.2	0
MMRN1	0.92	26.2	0

RGS5	0.91	22.9	0
UACA	0.91	39	0
WARS	0.91	46.9	0
GIMAP7	0.9	63.7	0
MMRN2	0.9	38.1	0
LMCD1	0.9	38.9	0
LMO2	0.9	43.7	0
CD36	0.89	31.1	0
PPFIBP1	0.89	37.7	0
S100A13	0.89	68.2	0
COX7A1	0.89	42.2	0
HEG1	0.88	38.7	0
CYYR1	0.88	37.2	0
LAPTM4A	0.88	79.3	0
BMPR2	0.88	42	0
TMEM204	0.87	40.1	0
GPIHBP1	0.87	26.4	0
AKAP12	0.87	21	0
CXorf36	0.87	37.7	0
TGFBR2	0.87	52.1	0
C8orf4	0.86	42	0
PLK2	0.86	38.6	0
CX3CL1	0.86	24.9	0
CD151	0.85	66.3	0
PTMS	0.85	66.4	0
VIM	0.85	97.1	0
SEC14L1	0.84	45.5	0
WWTR1	0.84	41.9	0
PHACTR2	0.83	46.9	0
TINAGL1	0.83	36	0
FAM43A	0.82	38.3	0
ANGPT2	0.82	21.1	0
MYH9	0.82	63	0
GPR146	0.82	34.1	0
MARCKS	0.82	55.8	0
FRY	0.81	32.6	0
GAS6	0.8	42.8	0
WBP5	0.8	48	0
LUZP1	0.8	46.3	0
BTNL9	0.8	22.5	0
LEPR	0.79	29.3	0
ITGB1	0.79	67.4	0
FSCN1	0.79	37.1	0
MYCT1	0.79	38.1	0
IFITM2	0.79	88.2	0
SERPINH1	0.79	42.4	0

KANK3	0.78	35.9	0
RAB13	0.78	55.7	0
SOX18	0.78	32.1	0
LEPROT	0.78	54.5	0
CDC37	0.77	68.8	0
GIMAP8	0.77	31.8	0
KCTD12	0.77	44.1	0
FENDRR	0.77	23.3	0
MEIS2	0.76	31	0
TJP1	0.76	40.5	0
MCAM	0.76	30.5	0
HLA-B	0.75	99.1	0
DLC1	0.75	35.2	0
NGFRAP1	0.75	56.9	0
ESM1	0.75	13.1	0
CRIM1	0.75	35.5	0
ERG	0.75	34.7	0
SOX7	0.75	29.2	0
GALNT18	0.75	32.8	0
GJA1	0.74	29.5	0
FOXF1	0.74	23.6	0
NFIA	0.74	41.7	0
HEY1	0.74	24.7	0
GSN	0.73	58	0
HTRA1	0.73	35	0
APOL3	0.73	40.4	0
ADGRL2	0.73	27.7	0
LAMA4	0.73	32.7	0
VIPR1	0.73	19.8	0
ARL2	0.73	54.5	0
TMEM255B	0.72	35.6	0
NPR3	0.72	23.5	0
CCL14	0.72	24.2	0
SOX4	0.72	50.5	0
KIAA1462	0.71	29.8	0
LIMS2	0.71	32.2	0
TSPAN12	0.71	28.4	0
GJA5	0.71	12.6	0
PKIG	0.71	42.4	0
RBP1	0.7	32.1	0
ITGA6	0.7	32.2	0
ECE1	0.7	38	0
LXN	0.69	31.4	0
ZNF503	0.69	30.8	0
GATA2	0.69	32	0
IL6ST	0.69	44.3	0

PROCR	0.68	33.4	0
RASIP1	0.68	31.6	0
PVRL2	0.68	40.5	0
LRRC32	0.68	23.2	0
FCGRT	0.68	70.1	0
PRKCDBP	0.68	34	0
KDR	0.68	27.9	0
LHFP	0.68	31.4	0
EFNA1	0.67	38	0
RCN1	0.67	40.8	0
SEMA6A	0.67	27.5	0
S1PR1	0.67	36	0
MYL12B	0.67	89.1	0
SOCS2	0.67	30.7	0
FERMT2	0.66	32.5	0
SCARF1	0.66	29.3	0
PLPP3	0.65	32.8	0
ITGA5	0.65	35	0
NR2F2	0.64	27.5	0
LAMB2	0.64	32.2	0
NDRG2	0.64	31.4	0
TEK	0.64	26.6	0
TMEM88	0.63	24.2	0
PLS3	0.63	34.8	0
MAOA	0.63	26.2	0
GIMAP1	0.63	44.9	0
ITGA1	0.63	25.3	0
MYL9	0.62	34.8	0
ELK3	0.62	36.3	0
RAPGEF5	0.62	27.6	0
HHEX	0.61	33.3	0
IL3RA	0.61	27.4	0
EPB41L2	0.6	29	0
PCDH12	0.6	22.8	0
DPYSL3	0.6	27.2	0
CYTL1	0.6	20.1	0
MEF2C	0.6	43.7	0
RGS3	0.59	27.7	0
RHOJ	0.59	28.9	0
PLXND1	0.59	32.7	0
NES	0.59	23	0
RGL2	0.59	34.5	0
RPLP1	-0.59	97.4	0
RPS15A	-0.6	94.9	0
RPS2	-0.6	92.5	0
RPL23A	-0.6	94.9	0

RPL41	-0.61	97.2	0
RPLP2	-0.63	97.6	0
RPL28	-0.73	95.2	0
RPSA	-0.75	83.5	0
RPL39	-0.75	91.4	0
RPS29	-0.89	92.1	0
CYBA	-0.91	56.9	0
RPS27	-0.99	97	0
LAPTM5	-1	36.3	0
CD53	-1.05	6.7	0
CD44	-1.07	13	0
LSP1	-1.08	8	0
PTPRC	-1.12	7.6	0
RAC2	-1.12	7.5	0
HCST	-1.26	10.7	0
CD48	-1.26	5.8	0
CD37	-1.5	8.9	0
CORO1A	-1.53	7.8	0
CD52	-1.7	24.5	0
HLA-C	0.7	98.5	5.95E-306
EPHX1	0.66	41.5	2.35E-303
FDPS	0.72	55.8	9.17E-300
GUK1	0.63	83.5	9.63E-296
CXCR4	-1.61	18.4	1.42E-289
PABPC1	-0.67	64.9	4.75E-288
CTNND1	0.64	38	3.22E-286
HLA-A	0.59	99.3	1.32E-285
INSR	0.86	23.9	1.03E-282
TIMP1	0.61	73.4	1.21E-279
TSPAN4	0.69	44	1.97E-279
LTB	-1.72	6.1	3.51E-279
ITGB2	-0.98	8.7	3.24E-269
RHOB	0.9	57.8	1.01E-264
CLIC4	0.62	36.4	3.60E-262
RGS10	-0.83	7.6	1.45E-260
GNAI2	0.71	64	2.52E-260
RALB	0.59	38.3	1.71E-258
CCND1	0.76	38.7	6.81E-256
CARHSP1	0.71	52.4	1.37E-250
TRAC	-1.63	5.9	1.26E-246
CD320	0.6	36.9	6.78E-246
TPM4	0.68	64	2.18E-245
DSTN	0.59	72.2	8.52E-245
GPSM3	-0.82	19.9	6.49E-243
GIMAP4	0.59	55.6	5.70E-242
FOS	0.77	83.1	1.39E-241

VAMP3	0.63	42.5	1.52E-239
CYB5R3	0.67	48	1.79E-239
RPS21	-0.61	79.5	4.93E-238
RALA	0.79	47.9	7.36E-236
YWHAE	0.63	66.4	5.55E-235
NRP1	0.61	30.4	7.68E-235
EVI2B	-0.83	5.2	5.12E-233
LDHB	-0.81	35.4	3.13E-232
UCP2	-0.83	9.4	1.16E-231
DUSP6	0.64	44.2	4.03E-231
TSTD1	-0.78	4.6	9.59E-231
FYB	-0.95	5.7	2.11E-230
RPS10	-0.61	79.4	6.49E-230
PRPSAP1	0.61	33.4	2.97E-228
STK17B	-0.82	5.3	7.84E-228
CYTIP	-0.95	3.8	7.96E-228
LITAF	-0.78	19.2	4.03E-227
EID1	0.6	73.3	4.59E-226
CD3D	-1.39	4.8	1.54E-225
ACAP1	-0.95	3.2	1.10E-224
PRMT1	0.68	50.2	8.22E-223
CD69	-1.45	5.6	4.57E-222
ACTN4	0.65	52.3	3.91E-215
EGR1	0.92	52.3	2.52E-214
IL2RG	-0.88	5.1	2.89E-212
LCP1	-0.77	6.3	2.87E-208
TRBC2	-1.3	5.2	1.57E-207
RNASET2	-0.75	16.9	1.15E-203
S100A4	-0.86	50.8	1.01E-201
TUBA1A	0.75	63.4	7.55E-201
RGS1	-1.54	7.6	8.40E-201
MAGED2	0.6	42.3	5.92E-200
CYR61	0.59	27.7	5.52E-199
LIMD2	-0.91	15.6	1.39E-198
SH3BGRL3	-0.69	73	2.13E-198
ALOX5AP	-1.1	12.3	5.54E-196
CD3E	-1.13	4.1	1.02E-195
CD2	-1.15	3.7	1.47E-189
IER2	0.75	76.6	1.60E-184
CFAP20	0.62	33	7.32E-184
SFTPC	-1.62	56.2	1.38E-182
VWA1	0.61	24	2.90E-182
CELF2	-0.62	5.2	1.83E-181
SERTAD1	0.78	46.4	3.36E-177
EZR	-0.69	15.1	1.05E-174
IFNGR1	0.7	42.2	3.25E-172

MYO6	0.59	30.6	6.02E-170
FAM49B	-0.61	14.8	2.49E-167
ARGLU1	0.78	62.2	5.53E-167
LAP3	0.65	44.8	5.38E-166
EFEMP1	0.82	19.6	1.12E-164
ZFP36	0.79	77.9	2.97E-163
ARHGAP18	0.71	37.6	5.99E-160
VAMP8	-0.62	39	3.19E-159
LCK	-0.84	3.3	1.88E-154
CD7	-1.05	4.8	8.62E-154
SPINT2	-0.78	10.7	7.58E-151
RHOH	-0.78	2.7	5.93E-150
TFF3	1.08	18.1	1.70E-149
FOSB	0.67	65.5	1.88E-148
EVL	-0.72	24.6	2.12E-147
GPR183	-0.96	5.2	6.17E-146
TBC1D10C	-0.67	3.4	1.69E-145
HCLS1	-0.59	8.6	2.36E-144
DUSP2	-1.03	10	1.37E-143
PLAC8	-0.84	4.5	1.83E-141
FABP5	1.03	49.7	5.08E-141
CAPG	-0.79	12.5	3.41E-138
EMP3	-0.6	35.6	5.10E-138
COTL1	-0.73	32.5	2.14E-137
SPRY1	0.59	22.9	2.27E-136
TRBC1	-1.31	4.1	2.91E-136
SAMSN1	-0.72	5	1.07E-135
PLP2	-0.6	23.9	5.72E-135
IFIT3	0.64	28.1	1.52E-134
TRAF3IP3	-0.67	1.8	1.23E-133
BIN2	-0.6	2.6	2.18E-132
STK17A	-0.68	14	5.44E-131
CRIP1	-0.61	10.1	3.70E-130
CTSC	-0.71	19.8	1.53E-128
GADD45B	0.91	55.4	1.15E-127
CCL5	-1.92	10.9	6.50E-125
RGS2	-0.79	10.5	1.38E-122
ZFAS1	-0.65	53.2	2.84E-122
CST7	-1.12	5.6	3.04E-121
SCGB1A1	-0.68	28.1	1.83E-119
CLEC2D	-0.68	4.7	2.58E-116
C1orf162	-0.71	8.2	5.99E-116
CD3G	-0.7	1.9	2.76E-112
FABP4	0.81	24.7	1.48E-105
CD27	-0.65	1.2	2.42E-104
CTSS	-0.68	26.5	2.35E-103

TSC22D3	-0.71	43.9	2.64E-103
LAT	-0.62	3.5	3.13E-101
GZMA	-1.24	4.3	1.01E-100
PTPN7	-0.59	2.8	1.19E-100
CD247	-0.7	2.6	3.46E-99
IRF1	0.81	57.3	6.51E-99
ARL4C	-0.62	10.4	2.18E-97
CCL4	-1.71	14.4	2.61E-97
ATP1B1	-0.61	6.8	8.53E-93
CCR7	-0.61	0.7	3.77E-89
SELL	-0.61	3.5	4.15E-88
IGKC	-1.84	32.6	7.65E-88
MGST1	-0.84	7.7	6.58E-86
GZMM	-0.64	3.9	8.09E-85
ICAM1	0.69	34.1	8.02E-82
AREG	-0.74	4.9	1.01E-81
AIF1	-0.96	11	1.05E-81
TNFAIP3	-0.66	18.5	4.90E-79
KRT19	-1.16	4.8	5.93E-79
LST1	-0.73	8.5	1.71E-76
CTSW	-0.78	3.3	5.18E-76
FCGR3A	-0.75	6.4	5.97E-76
HPGD	0.82	27.9	6.10E-76
C15orf48	-0.8	5.3	3.24E-75
RGCC	0.73	52.8	2.12E-73
CD79A	-0.99	1.2	6.09E-73
S100A9	-1.42	18.2	8.37E-73
KLRB1	-1.17	4.3	2.73E-70
IGLC2	-2.36	15.8	3.44E-70
CLDN4	-0.7	3.5	1.42E-69
CCL4L2	-1.39	8.3	1.96E-68
CCL3	-1.49	10.9	2.11E-66
CCL3L3	-1.12	5.4	7.81E-66
BTG1	-0.63	83	1.63E-64
CPE	0.61	16.8	2.45E-64
FCER1G	-1.06	19.2	2.65E-64
TYROBP	-1.23	26.6	1.08E-63
SERPINA1	-0.82	12.2	2.26E-62
ELF3	-0.77	4.6	4.92E-62
ACP5	-0.59	11.6	9.82E-62
MUC1	-0.69	5.3	1.53E-61
NKG7	-1.56	12.1	6.82E-61
MS4A6A	-0.64	4.6	1.10E-60
AGR2	-1.04	4.9	3.14E-60
WFDC2	-1.04	7.1	3.81E-57
MS4A1	-0.67	0.5	6.85E-57

CEACAM6	-0.59	2.3	1.09E-56
KRT7	-0.79	4.6	1.28E-56
SLC34A2	-0.63	2.4	5.88E-56
PRF1	-0.69	2.4	1.77E-54
S100A14	-0.59	2.5	2.00E-53
GZMK	-0.91	1.7	1.97E-52
SFTA2	-0.68	5.4	1.37E-51
CD68	-0.79	12.6	6.41E-51
GZMH	-0.93	4	3.67E-49
FTH1	-0.83	99.3	1.26E-47
NAPSA	-0.89	6.7	5.09E-45
S100A8	-0.92	7.5	9.40E-41
APOE	-1.42	22.7	2.05E-40
C1QC	-0.93	8.6	2.34E-37
GZMB	-1.04	5.9	1.42E-36
CFD	-0.81	10.4	2.43E-36
KLRD1	-0.6	2.4	8.61E-36
IGHG3	-1.47	12.5	9.27E-35
KRT8	-0.68	10.8	4.52E-33
IGHM	-1.16	4.2	5.09E-33
IL1B	-0.64	5.5	2.08E-32
SPP1	-1.79	5.8	6.95E-30
HLA-DQB1	-0.63	42.2	5.76E-29
G0S2	-0.62	6.2	1.17E-28
SCGB3A1	-1.17	24.6	1.58E-27
JCHAIN	-1.22	5.3	3.22E-25
HLA-DQA1	-0.65	31.1	4.98E-25
IGHG4	-1.19	12.5	5.21E-23
GNLY	-1.46	8.2	2.54E-22
CTSD	-0.74	55.4	5.79E-22
IGHG1	-1.67	10.2	2.06E-21
CXCL8	-0.79	18.6	4.01E-21
CREM	-0.67	28	8.55E-19
S100A11	-0.6	74.6	1.01E-18
KRT18	-0.74	16.4	1.21E-15
IGHA1	-1.43	17.5	1.68E-14
MT1X	-0.63	18	8.58E-13
LYZ	-1.09	25.1	1.23E-12
CSTB	-0.63	64.3	2.35E-12
IGLC3	-1.74	12.6	7.11E-11
CTSB	-0.66	40.4	5.24E-10
SFTPB	-1.18	14.6	1.24E-09
FTL	-1.15	99.5	3.78E-09
APOC1	-1.22	31.3	1.93E-06
SCGB3A2	-1.05	8.3	4.82E-06

Table S3 13 differentially expressed endothelial cell immune-related genes.

ID	HR	HR.95L	HR.95H	pvalue
RAB11A	1.4817725	1.1110645	1.976167	0.0074306
TNFRSF1A	1.5872882	1.2183479	2.067951	0.0006187
CXCR4	0.8472121	0.7235127	0.992060	0.0394991
YWHAE	1.5923339	1.1739897	2.159752	0.0027764
PRMT1	1.4307283	1.0895412	1.878757	0.0099680
CD37	0.8490144	0.7384717	0.976104	0.0214602
CYTIP	0.8352127	0.7176706	0.972006	0.0199725
ADM	1.2985825	1.1593635	1.454519	6.31E-06
ITGB1	1.5315501	1.238890	1.893344	8.15E-05
CD48	0.8269481	0.7108363	0.962026	0.0138369
AREG	1.0873027	1.0100324	1.170484	0.0260563
LST1	0.8745152	0.7676533	0.996252	0.0437560
PTPRC	0.8499128	0.7494751	0.963810	0.0112629

Table S4 8 differentially expressed endothelial cell immune-related genes.

Gene	Coef
TNFRSF1A	0.116157
CXCR4	-0.068285
YWHAE	0.170979
PRMT1	0.001031
ADM	0.196255
ITGB1	0.240534
AREG	0.060144
PTPRC	-0.149539

Table S5 Univariate and multifactorial Cox regression.

ID	HR	HR.95L	HR.95H	P-value
**Univariate Cox regression**				
Age	1.007642	0.992402	1.023117	0.327532
Gender	1.113087	0.828925	1.494663	0.476226
Stage	1.639217	1.425885	1.884466	3.72E-12
RiskScore	3.426259	2.462393	4.767417	2.74E-13
**Multifactorial Cox regression**				
Age	1.016364	1.001023	1.03194	0.036468
Gender	0.862716	0.638771	1.165173	0.335534
Stage	1.515282	1.309332	1.753626	2.46E-08
RiskScore	3.085045	2.168399	4.389185	3.79E-10

Table S6 Antineoplastic drug sensitivity information.

Drugs	P-value
**More sensitive in high-risk group**	
ABT737	1.47E-12
Axitinib	1.82E-10
AZD6482	1.68E-09
AZD8055	2.90E-05
BMS.754807	4.10E-16
Doramapimod	2.22E-20
GSK269962A	8.38E-11
JAK1_8709	0.000532112
KU.55933	0.000502903
Mitoxantrone	0.007175219
PF.4708671	6.18E-15
PRT062607	1.78E-11
Ribociclib	1.62E-22
SB216763	2.02E-21
SB505124	3.40E-08
Selumetinib	0.000205119
Sepantronium.bromide	0.001217737
Sinularin	6.86E-05
Tozasertib	0.006395235
Venetoclax	0.000122756
WEHI.539	0.002404234
**More sensitive in low-risk group**	
AGI.5198	1.21E-05
Alisertib	0.000689507
Alpelisib	0.000410616
AT13148	0.02462595
AZD1332	5.44E-05
AZD3759	0.026505466
AZD4547	0.004919102
AZD6738	4.39E-09
AZD7762	0.000403147
BDP.00009066	0.000301072
BI.2536	1.83E-14
BMS.345541	3.58E-07
BMS.536924	1.47E-07
BPD.00008900	0.00059533
Buparlisib	0.010095547
Camptothecin	2.60E-05
Cediranib	2.00E-05
Cisplatin	2.22E-10
Crizotinib	1.95E-05
Cytarabine	0.000106045
Dactinomycin	0.028639749

Dactolisib	0.000423665
Daporinad	0.003105173
Dasatinib	1.55E-06
Docetaxel	4.76E-07
Elephantin	0.019193813
Epirubicin	0.001511734
EPZ004777	0.006731955
ERK_2440	0.000519071
ERK_6604	1.40E-09
Erlotinib	4.69E-05
Foretinib	6.60E-09
Fulvestrant	0.002373355
Gallibiscoquinazole	4.14E-05
GDC0810	0.000730272
Gefitinib	0.000139514
Gemcitabine	0.000178829
GSK2578215A	0.011394602
GSK2606414	4.07E-05
GSK343	2.33E-07
IAP_5620	0.018973772
IGF1R_3801	2.53E-06
Ipatasertib	0.041971197
Irinotecan	0.026464221
JQ1	0.003707703
Lapatinib	0.016613318
LCL161	5.17E-08
Leflunomide	1.70E-07
Luminespib	0.002740754
Mirin	0.021346923
MK.1775	3.79E-09
MK.2206	0.033405583
MK.8776	0.040347308
NVP.ADW742	0.047925685
Osimertinib	0.007896412
OTX015	0.000423999
Paclitaxel	7.56E-08
PAK_5339	0.005535673
PD0325901	0.000119168
Pevonedistat	6.32E-08
Picolinici.acid	0.001267006
Pictilisib	0.014369038
PRIMA.1MET	0.000405002
Sabutoclax	0.034116973
Sapitinib	0.002730115
Savolitinib	1.62E-07
SCH772984	9.89E-19
Staurosporine	2.46E-05
Talazoparib	1.88E-05
Taselisib	0.000201222

Telomerase.Inhibitor.IX	1.29E-07
Trametinib	0.002520674
Ulixertinib	7.04E-07
ULK1_4989	0.000951144
VE821	0.000497244
Vinblastine	0.000192432
Vincristine	0.013294089
Vinorelbine	0.00022954
VSP34_8731	0.029443523
VX.11e	5.94E-11
Wee1.Inhibitor	1.34E-06
5.Fluorouracil	8.28E-13
YK.4.279	2.11E-05
ZM447439	1.60E-07
**Similar sensitivity in the high- and low- risk groups**	
Acetalax	0.2326794
Afatinib	0.0957559
Afuresertib	0.4862247
AGI.6780	0.4877444
AMG.319	0.3597699
AZ6102	0.1199853
AZ960	0.36232
AZD1208	0.0561956
AZD2014	0.7973605
AZD5153	0.5350937
AZD5363	0.924888
AZD5438	0.841268
AZD5582	0.787081
AZD5991	0.8321859
AZD8186	0.5190625
BIBR.1532	0.9581747
Bortezomib	0.6850406
Carmustine	0.9504474
CDK9_5038	0.8772499
CDK9_5576	0.7971293
Cyclophosphamide	0.885505
CZC24832	0.3616814
Dabrafenib	0.130742
Dihydrorotenone	0.4790411
Dinaciclib	0.1365447
Eg5_9814	0.4992234
Entinostat	0.1751082
Entospletinib	0.1489423
EPZ5676	0.5767819
Fludarabine	0.142397
GNE.317	0.0546721
GSK1904529A	0.917186
GSK591	0.3937983
I.BET.762	0.0526958

I.BRD9	0.115013
Ibrutinib	0.7271487
IRAK4_4710	0.6035593
IWP.2	0.1024592
JAK_8517	0.7950211
KRAS..G12C..Inhibitor.12	0.1918782
LGK974	0.7806923
Linsitinib	0.6600351
LJI308	0.3120205
LY2109761	0.8497275
MG.132	0.2009068
MIM1	0.2557499
MIRA.1	0.0964829
ML323	0.1745302
MN.64	0.1729958
Navitoclax	0.1863637
Nelarabine	0.4942316
Nilotinib	0.6356304
Niraparib	0.0593124
NU7441	0.2137937
Nutlin.3a....	0.450531
Obatoclax.Mesylate	0.0785511
OF.1	0.8166106
Olaparib	0.7810241
OSI.027	0.5101791
Oxaliplatin	0.3958192
P22077	0.1018232
Palbociclib	0.189822
PCI.34051	0.1089938
PD173074	0.1594571
PFI3	0.5755405
PLX.4720	0.6648728
Podophyllotoxin.bromide	0.2461126
Pyridostatin	0.302257
Rapamycin	0.5689415
RO.3306	0.4869842
